# Integration of microphysiological systems with computational and digital twin modeling for pharmaceutical development: a systematic review

**DOI:** 10.3389/jpps.2026.16454

**Published:** 2026-09-04

**Authors:** Marzieh Soheili, Behdad Gilzad Kohan, Leila Rastegar, Yousef Moradi, Hamed Gilzad Kohan

**Affiliations:** 1 College of Pharmacy, Western New England University, Springfield, MA, United States; 2 School of Public and International Affairs, Princeton University, Princeton, NJ, United States; 3 School of Medicine, Jahrom University of Medical Sciences, Jahrom, Fars, Iran; 4 Health Metrics and Evaluation Research Center, Research Institute for Health Development, Kurdistan University of Medical Sciences, Sanandaj, Iran

**Keywords:** digital twin, drug development, in vitro-to-in vivo extrapolation, IVIVE, microphysiological systems

## Abstract

**Background:**

The pharmaceutical industry faces a productivity crisis, with only ∼10% of candidates reaching approval and development costs exceeding $1-2 billion per drug. Traditional preclinical models, particularly animal studies, show limited predictive validity for human outcomes. Microphysiological systems (MPS), including organ-on-chip devices, integrated with digital twin computational modeling, offer a promising route to improve translational prediction, yet no systematic assessment exists.

**Objectives:**

To identify, evaluate, and synthesize the published literature on integrating MPS with digital twin computational modeling for pharmaceutical development.

**Methods:**

Following PRISMA 2020 guidelines (PROSPERO: CRD420251274941), PubMed and Europe PMC were searched from January 2010 to December 2025. Studies were included if they integrated MPS platforms with computational models (PBPK, QSP, IVIVE, PK/PD, or machine learning). Two-stage automated screening with independent verification by two researchers was applied. Data extraction captured organ systems, model types, platforms, validation approaches, pharmacokinetic parameters, software, and economic considerations.

**Results:**

Of 2,044 records, 123 studies met inclusion criteria. Publication activity grew exponentially (CAGR ∼35%), with 38.2% published in 2024–2025. Liver was the most represented organ (30.9%), followed by vasculature (19.5%), gut (18.7%), and immune components (17.1%). PBPK was the predominant computational approach (18.7%), followed by PK (12.2%), IVIVE (12.2%), and machine learning (9.8%). Commercially, Emulate led (55.8%), then Mimetas (23.3%) and CN Bio (19.2%); custom/academic platforms comprised 74.2%. Validation was robust, with 95.8% discussing clinical data comparisons and 77.5% reporting average fold error. Only liver-PBPK and liver-PK qualified as established combinations (>5 studies), with many organ-model pairings unexplored. Multi-organ systems showed accelerating adoption after 2021. Economic themes were discussed qualitatively, but formal health economic analyses were absent.

**Conclusion:**

MPS-digital twin integration has matured from an emerging concept into a research paradigm with demonstrated translational value. Validation practices are strong and aligned with regulatory expectations, supported by the FDA Modernization Act 2.0 and the ISTAND program’s acceptance of Liver-Chip technology. Liver-focused applications are the most mature and should serve as a template for expansion. Priority gaps include kidney, lung, and brain MPS-computational integration, multi-organ systems, and rigorous health economic analyses. Continued standardization and regulatory engagement will be essential to realize this approach’s potential.

## Introduction

### The pharmaceutical R&D productivity crisis

The pharmaceutical industry confronts a profound and persistent productivity crisis in drug development. Despite remarkable advances in scientific understanding, technological capabilities, and managerial approaches over the past six decades, the number of new drugs approved per billion U.S. dollars invested in research and development has declined approximately 80-fold in inflation-adjusted terms, a phenomenon termed “Eroom’s Law” [1]. This exponential decline in efficiency, roughly halving every 9 years since 1950, has transformed pharmaceutical R&D into one of the most challenging investment environments in the technology sector [1, 2].

The most comprehensive analyses of clinical development success rates reveal that only approximately 10% of drug candidates entering Phase I clinical trials ultimately receive regulatory approval [3–5]. Phase II and Phase III failures represent particularly costly setbacks, with safety-related failures accounting for a substantial proportion of late-stage attrition [6, 7]. Conservative estimates suggest that developing a new drug requires investments exceeding $1-2 billion and timelines spanning 10–15 years [8, 9], with approximately 75% of these costs attributable to failures, resources expended on candidates that demonstrated apparent promise in preclinical testing but subsequently proved ineffective, unsafe, or commercially non-viable in human trials [10, 11].

### Translational failure and limitations of preclinical models

A fundamental driver of pharmaceutical attrition is the limited predictive validity of preclinical models, particularly animal studies that have served as regulatory requirements for decades. Systematic reviews comparing treatment effects in animal experiments with subsequent clinical trial outcomes have consistently demonstrated poor concordance [12, 13]. The value of animal experiments for predicting treatment effectiveness in humans has remained controversial, with recurrent failures of interventions that appeared promising in animal models to translate successfully to clinical benefit [13, 14]. Preclinical animal models (rodent and non-rodent) have limited predictive power for human drug-induced liver injury, with concordance between animal toxicity outcomes and clinical DILI observed in only approximately 50–60% of cases due to species-specific differences in drug handling and toxicological responses [15].

Multiple factors contribute to translational failure. Species-specific differences in drug metabolism, immune responses, pharmacokinetics, and disease pathophysiology render animal data unreliable predictors of human outcomes [16, 17]. Methodological limitations in animal studies, including inadequate randomization, lack of blinding, and publication bias favoring positive results, further compromise the reliability of preclinical evidence [13]. Furthermore, although genetically engineered animal models of human disease are phenotypically similar, they often operate through distinct molecular and cellular mechanisms, potentially directing researchers toward therapeutic targets that lack clinical relevance [17]. The disconnect between preclinical model predictions and clinical outcomes has been characterized as a primary cause of the pharmaceutical productivity crisis [18, 19].

### Microphysiological systems: a new paradigm

The recognition of these limitations has catalyzed intensive efforts to develop alternative preclinical platforms with improved human relevance. Among these approaches, microphysiological systems (MPS), including organ-on-a-chip (OoC) devices, have emerged as up-and-coming technologies. MPS are complex, multicellular *in vitro* culture systems designed to recapitulate the physiological properties of specific organs or tissues that govern their function [17, 20]. The International Consortium for Innovation and Quality (IQ) MPS Affiliate defines MPS as going beyond traditional 2D culture models by incorporating multicellular environments within biopolymer or tissue-derived matrices, 3D structures, mechanical cues such as stretch or perfusion, and primary or stem cell-derived cells [21].

The foundational demonstration of this approach came in 2010 when Huh and colleagues described a biomimetic microdevice that reconstituted the functional alveolar-capillary interface of the human lung, reproducing complex integrated organ-level responses to bacteria, inflammatory cytokines, and nanoparticles. This seminal work established that mechanically active organ-on-a-chip microdevices incorporating physiological breathing motions could recapitulate tissue-to-tissue interfaces critical to organ function with unprecedented fidelity. The study demonstrated that cyclic mechanical strain accentuated toxic and inflammatory responses, highlighting the importance of dynamic mechanical cues that cannot be reproduced in conventional static culture systems [22].

The subsequent decade witnessed extraordinary advancement in MPS technology, with platforms now available for virtually every major organ system [17, 20]. Modern organ chip platforms incorporate multiple cell types, including parenchymal cells, endothelial barriers, immune cells, and supporting stromal elements, enabling recapitulation of complex tissue architectures and cellular interactions. Advanced multi-organ systems link mature human heart, liver, bone, and skin tissue niches through recirculating vascular flow, separated by selectively permeable endothelial barriers, enabling unprecedented recapitulation of pharmacokinetic and pharmacodynamic profiles and the identification of early biomarkers of drug toxicity [23]. Beyond modeling homeostatic organ function, MPS platforms have been adapted to model complex tumor-immune microenvironments, with 3D microfluidic culture systems enabling the *ex vivo* evaluation of patient-derived organotypic tumor spheroids that retain autologous immune cells and respond to immune checkpoint blockade [24].

### Computational modeling in pharmaceutical development

Parallel to experimental advances, computational modeling approaches have become integral to modern drug discovery and development. Physiologically based pharmacokinetic (PBPK) modeling represents the body as a network of individual organs connected via arterial and venous blood flow, enabling mechanistic prediction of drug absorption, distribution, metabolism, and excretion (ADME) from physicochemical properties and *in vitro* data [25–27]. These quantitative mechanistic frameworks employ in vitro-to-in vivo extrapolation (IVIVE) techniques to scale drug-specific parameters, facilitating prediction of human pharmacokinetics and informing first-in-human dose selection, drug-drug interaction assessment, and special population analyses.

Beyond PBPK modeling, quantitative systems pharmacology (QSP) approaches integrate drug mechanism, disease biology, and patient physiology into comprehensive mathematical frameworks capable of predicting therapeutic outcomes [28, 29]. Machine learning and artificial intelligence methods are increasingly applied to identify patterns in high-dimensional biological data. However, all computational approaches face inherent limitations in capturing organ-specific differentials in drug behavior resulting from complex cellular uptake, transport, metabolism, and tissue binding that vary across organ systems and between individuals.

The integration of MPS-derived experimental data with computational models offers a compelling solution to these challenges. Organ chip devices that recapitulate tissue-tissue interfaces, vascular perfusion, and organ-level functionality can provide the mechanistic parameters needed for more accurate PBPK/PD modeling while enabling direct assessment of drug efficacy and toxicity in human cellular contexts [30]. When multiple organ chips are linked via endothelium-lined vascular channels, they overcome many limitations inherent to single-tissue systems, enabling physiologically relevant in vitro-to-in vivo extrapolation of whole-body drug responses [31].

### The digital twin paradigm

The concept of digital twins, virtual representations of physical systems enabling real-time simulation, prediction, and optimization, has gained substantial traction in pharmaceutical development [32, 33]. In the context of drug development and clinical trials, digital twins are virtual representations of systems of varying complexity, ranging from individual cells to entire human patients, enabling *in silico* simulations and experiments. There is a bidirectional information flow between a biological entity and its digital twin, in which the digital twin is initialized with measurements from the biological entity and returns predictions or simulation results from virtual experiments that inform decision-making about the biological entity [32]. By creating virtual models of biological systems, researchers can better predict which drug candidates are likely to succeed in clinical studies, ultimately reducing their dependence on preclinical animal studies [34]. This integration enables bidirectional information flow: MPS experiments generate mechanistic parameters that inform and calibrate computational models, while model predictions guide experimental design and identify knowledge gaps that require further investigation. The resulting synergy promises more accurate prediction of human drug responses than either experimental or computational approaches can achieve independently [30, 31].

Multiple computational methodologies can be integrated with MPS platforms to address different aspects of drug development [35, 36]. PBPK models provide whole-body pharmacokinetic predictions essential for dose selection and exposure estimation, while integrating MPSs into PBPK modeling enables more reliable and relevant parameter values with a more accurate representation of human physiology [36]. The combination of PBPK modeling and organ-on-a-chip is believed to provide a powerful new tool for drug development, with the potential to replace animal testing [35]. QSP models capture relationships between disease biology and drug mechanisms to predict efficacy [28, 29]. Machine learning approaches identify patterns in complex datasets and enable predictions where mechanistic understanding remains incomplete [37]. Mechanistic models describe specific biological processes at molecular, cellular, or tissue scales. The selection and combination of modeling approaches depend on the particular application, available data, regulatory context, and scientific questions under investigation [30, 35].

### Regulatory and economic context

The regulatory landscape is evolving rapidly to accommodate these technological advances. The FDA Modernization Act 2.0, signed into law in December 2022, eliminates the federal mandate requiring animal testing for FDA-approved drugs, which has been in effect since 1938. This landmark legislation defines “nonclinical tests” to include tests conducted *in vitro*, *in silico*, or in chemico, explicitly citing cell-based assays, organ chips, and microphysiological systems, and computer modeling as acceptable alternatives [38]. In April 2025, the FDA announced its Roadmap to Reducing Animal Testing in Preclinical Safety Studies, outlining a phased approach to encourage the submission of data from New Approach Methodologies (NAMs), initially targeting monoclonal antibodies before expanding to other biologics and new chemical entities [39]. The FDA’s ISTAND (Innovative Science and Technology Approaches for New Drugs) Program, introduced in 2020, explicitly listed microphysiological systems as qualifying technologies [40]. In September 2024, the first Organ-on-a-Chip, a Liver-Chip designed to predict drug-induced liver injury [40], was accepted into this pilot program.

The Innovation and Quality (IQ) consortium, a collaboration of pharmaceutical and biotechnology companies including AbbVie, AstraZeneca, Genentech, Janssen, Merck, Pfizer, and others, has played a pivotal role in establishing qualification frameworks for MPS technologies. The IQ MPS Affiliate, formed in 2018, has prepared organ-specific guidance documents defining performance criteria, characterization requirements, and contexts of use for liver, kidney, lung, gastrointestinal, cardiovascular, skin, and blood-brain barrier MPS models [21, 41]. These guidelines provide a three-stage benchmarking strategy with key performance metrics and compound test sets, helping MPS developers and pharmaceutical end-users identify models most valuable for specific safety risk assessment contexts [41]. Industry surveys conducted by the IQ MPS Affiliate in 2019 and 2021 documented growing pharmaceutical engagement with MPS technologies across safety, pharmacology, and ADME applications [42].

The economic implications of improved preclinical prediction are substantial. Systematic performance assessment of human Liver-Chips demonstrated 87% sensitivity and 100% specificity in predicting drug-induced liver injury across a blinded set of 27 hepatotoxic and non-toxic compounds recommended by the IQ consortium, substantially outperforming conventional preclinical models [11]. Notably, the Liver-Chip met IQ qualification guidelines, including the ability to distinguish between toxic drugs and their structurally related non-toxic analogs, a key criterion for regulatory acceptance [11, 41]. By comparison, traditional animal models and conventional *in vitro* assays typically achieve sensitivities of only 50–70% with lower specificity, contributing to both costly false negatives (hepatotoxic drugs advancing to clinical trials) and false positives (safe drugs inappropriately terminated) [43, 44].

Economic modeling based on these performance characteristics suggests that routine adoption of validated organ-chip technologies into preclinical workflows could generate over $3 billion annually for the pharmaceutical industry through increased R&D productivity and reduced late-stage attrition. This value derives from multiple sources: earlier termination of programs with hepatotoxic liabilities (reducing expensive late-stage failures), rescue of viable candidates incorrectly flagged as toxic by less specific assays, and acceleration of development timelines through more confident go/no-go decisions [11]. Beyond financial considerations, improved preclinical models offer benefits aligned with the 3Rs principles (Replacement, Reduction, and Refinement of animal use), addressing ethical concerns while enhancing the human relevance of preclinical data [20, 21].

### Knowledge gap and review rationale

Despite the compelling promise of integrating MPS platforms with computational modeling approaches, the field lacks a comprehensive synthesis of current evidence. While MPS have recently emerged to reconstitute the *in vivo* cellular microenvironment on *in vitro* platforms and provide reliable drug discovery testbeds, their adoption into drug discovery and evaluation processes still lags [36]. Individual studies have demonstrated proof of concept for specific applications across diverse organ systems, modeling methodologies, and pharmaceutical contexts [17, 45]. However, no systematic assessment has characterized the landscape of MPS-computational model integration, identified dominant approaches and emerging trends, mapped research gaps, or evaluated the maturity of different application areas. Broad adoption of MPS technology has been limited by translation gaps among platform developers, end-users, regulatory agencies, and the pharmaceutical industry [46]. The current challenges include a lack of rigorous standards for reproducibility and reliability, as well as practical difficulties in adoption within pharmaceutical research and industry settings [36]. Understanding the current state of this rapidly evolving field is essential for identifying research priorities, recognizing methodological best practices, informing regulatory science, and guiding strategic adoption decisions by the pharmaceutical industry [11, 20].

### Objectives

This systematic review aims to comprehensively identify, evaluate, and synthesize the published literature on integrating microphysiological systems with digital twin computational modeling for pharmaceutical development applications. Specific objectives include: (1) characterizing publication trends and the evolution of research in this field from 2010 to 2025; (2) identifying the organ systems, computational model types, and MPS platforms most commonly employed; (3) mapping the pharmaceutical applications addressed, including efficacy prediction, toxicity assessment, drug metabolism, and drug-drug interactions; (4) assessing validation approaches and pharmacokinetic parameters extracted from MPS experiments; (5) analyzing software tools and commercial platforms utilized; (6) evaluating integration methodologies linking experimental MPS data with computational models; (7) identifying research gaps and opportunities for future investigation; and (8) assessing the extent to which economic and translational considerations are addressed in the literature. Through this comprehensive assessment, we aim to provide an evidence-based foundation for advancing this promising approach to human-relevant preclinical drug evaluation.

## Methods

### Protocol and registration

This systematic review was conducted in accordance with the Preferred Reporting Items for Systematic Reviews and Meta-Analyses (PRISMA) 2020 guidelines [47]. The review protocol was registered prospectively with the International Prospective Register of Systematic Reviews (PROSPERO; registration number CRD420251274941; available from^1^). The methodology was designed to ensure reproducibility through fully automated computational approaches. The systematic review aimed to comprehensively identify, evaluate, and synthesize the current literature on integrating microphysiological systems (MPS) with digital twin computational modeling for pharmaceutical development applications.

### Eligibility criteria

#### Inclusion criteria

Studies were included if they met the following criteria: (1) Original research articles or methodological studies published in English between 1 January 2010 and 31 December 2025; (2) Studies describing microphysiological systems, including organ-on-chip devices, body-on-chip systems, tissue chips, or microfluidic cell culture platforms; (3) Studies using computational or mathematical modeling, including physiologically-based pharmacokinetic (PBPK) models, quantitative systems pharmacology (QSP) models, pharmacokinetic/pharmacodynamic (PK/PD) models, in vitro-to-in vivo extrapolation (IVIVE) methods, mechanistic models, or machine learning approaches; (4) Studies integrating MPS experimental data with computational models, including parameterization, calibration, validation, or predictive applications.

#### Exclusion criteria

Studies were excluded based on the following criteria: (1) Pure review articles, systematic reviews, meta-analyses, narrative reviews, or scoping reviews without original data; (2) Editorials, commentaries, letters to the editor, conference abstracts, book chapters, or errata/corrections; (3) Studies focusing exclusively on animal models or clinical trials without MPS components; (4) Studies describing MPS technology without computational modeling integration; (5) Studies presenting computational models without experimental MPS validation or parameterization.

### Information sources and search strategy

A comprehensive literature search was conducted on December 14, 2025, across two electronic databases: PubMed/MEDLINE and Europe PMC. The search strategy was designed to capture all relevant literature at the intersection of MPS technology and computational modeling for pharmaceutical applications.

The PubMed search employed a structured Boolean query restricted to Title/Abstract fields, combining MPS-related terms (including “microphysiological system,” “organ-on-chip,” “organ on chip,” “organ-on-a-chip,” “body-on-chip,” “tissue chip,” “multi-organ chip,” and organ-specific variants such as “liver-on-chip,” “heart-on-chip,” “kidney-on-chip,” “lung-on-chip,” “gut-on-chip,” “brain-on-chip,” and “skin-on-chip”) with computational modeling terms (including “PBPK,” “physiologically based pharmacokinetic,” “QSP,” “quantitative systems pharmacology,” “computational model,” “mathematical model,” “mechanistic model,” “pharmacokinetic model,” “digital twin,” “*in silico*,” “IVIVE,” “*in vitro* to *in vivo*,” “extrapolation,” and “scaling” combined with “pharmacokinetic”). The search was restricted to English-language publications from 1 January 2010 to 31 December 2025.

Europe PMC was searched using a complementary query adapted to the database’s syntax: “(microphysiological system OR organ-on-chip OR tissue chip OR body-on-chip) AND (PBPK OR computational model OR mathematical model OR digital twin OR IVIVE OR pharmacokinetic model)” with language and date filters applied (English language, 2010–2025). The Europe PMC search was designed to capture additional records through its broader indexing of European repositories, preprints, and full-text content. A maximum of 2,000 records were retrieved from Europe PMC due to API limitations.

The differing search sensitivities between databases (PubMed: high specificity with Title/Abstract restriction; Europe PMC: higher sensitivity with broader coverage) were intentional to balance precision with comprehensive retrieval. The subsequent two-stage screening process ensured that only studies meeting the predefined eligibility criteria were included, regardless of source database.

### Study selection process

The study selection process used a two-stage automated screening approach to ensure reproducibility and consistency. In the first stage (title and abstract screening), records were evaluated using a computational algorithm that assessed the presence of MPS-related terminology and computational modeling terminology. Studies were required to contain at least one term from each category to proceed to full-text assessment. A relevance score was calculated based on the frequency and specificity of matching terms, with studies scoring ≥20 points classified as “INCLUDE” and those scoring 1–19 points classified as “MAYBE” for additional review.

In the second stage (full-text screening), studies that passed the initial screening were subjected to a refined eligibility assessment using evidence-based criteria. Studies were evaluated for: (1) integration evidence demonstrating actual data flow between MPS experiments and computational models (including parameterization, calibration, validation, or predictive applications); (2) MPS platform evidence indicating specific experimental systems; and (3) computational model evidence describing model structure, software, or mathematical frameworks. Studies were classified as “INCLUDE_HIGH” (strong evidence across all three categories), “INCLUDE_MEDIUM” (evidence in integration plus one additional category), “INCLUDE_LOW” (integration evidence only), or “EXCLUDE_FULLTEXT” (insufficient integration evidence).

The automated screening results were independently verified by two researchers (MS and LR). All studies classified for inclusion (n = 123) were manually reviewed to confirm eligibility, and a random sample of excluded records was examined to identify potential false negatives. Disagreements were resolved through consensus discussion.

### Data collection and extraction

Data extraction was performed at two levels: abstract-level extraction for all included studies and full-text extraction for studies with available PDF documents. Abstract-level extraction captured bibliographic information (authors, year, journal, DOI, PMID), modeled organ systems, computational model types, MPS platform descriptions, cell types used, application domains, and validation approaches.

Full-text extraction used automated text mining of PDF documents converted to plain text. The extraction protocol captured: (1) Software and computational tools used (including PBPK platforms such as Simcyp, GastroPlus, and PK-Sim; programming environments such as MATLAB, R, and Python; and statistical software); (2) Pharmacokinetic parameters extracted from MPS (including intrinsic Clearance, hepatic Clearance, permeability coefficients, partition coefficients, protein binding, enzyme kinetic parameters Km and Vmax, and scaling factors); (3) Validation metrics reported (including fold error, average fold error [AFE], coefficient of determination [R^2^], root mean square error [RMSE], mean absolute error [MAE], and comparison with clinical data); (4) Commercial MPS platform identification; and (5) Culture conditions and experimental parameters. Additionally, full-text documents were screened for discussion of economic and translational considerations, including cost savings, time savings, animal reduction, and efficiency improvements.

The reliability of automated data extraction was validated through independent manual review by two researchers of a randomly selected subset of included studies. Extracted data elements were compared against automated results to ensure accuracy of the text-mining approach.

Automated text-mining frequencies represent detection of relevant terminology within full-text documents, which may include methodological discussions, literature references, or contextual mentions in addition to original experimental data. Consequently, high-frequency parameters (e.g., fraction unbound, validation approaches) reflect the centrality of these concepts in MPS-digital twin integration literature rather than universal experimental reporting.

### Data synthesis and analysis

Descriptive statistics were computed for all extracted variables. Publication trends were analyzed by year and model type. The distribution of organ systems, computational model types, MPS platforms, and application domains was summarized. Cross-tabulations were generated to identify patterns in organ-model combinations and research gaps. Temporal trends in multi-organ versus single-organ systems, validation approaches, and economic considerations were examined. All analyses were conducted in R (version 4.3) using the tidyverse, ggplot2, and related packages. Visualizations were generated with publication-quality formatting, consistent color schemes, and styling.

### Quality assessment and confidence classification

Studies were classified by confidence level based on the strength of evidence for MPS-computational model integration. High-confidence studies provided clear evidence of integration across all assessment domains (integration evidence, MPS platform description, and computational model specification). Medium-confidence studies presented integration evidence, along with partial evidence, in support of the domains. Low-confidence studies showed minimal evidence of integration. This classification system was used to weigh the reliability of findings in the synthesis.

## Results

### Study selection

**FIGURE 1:**
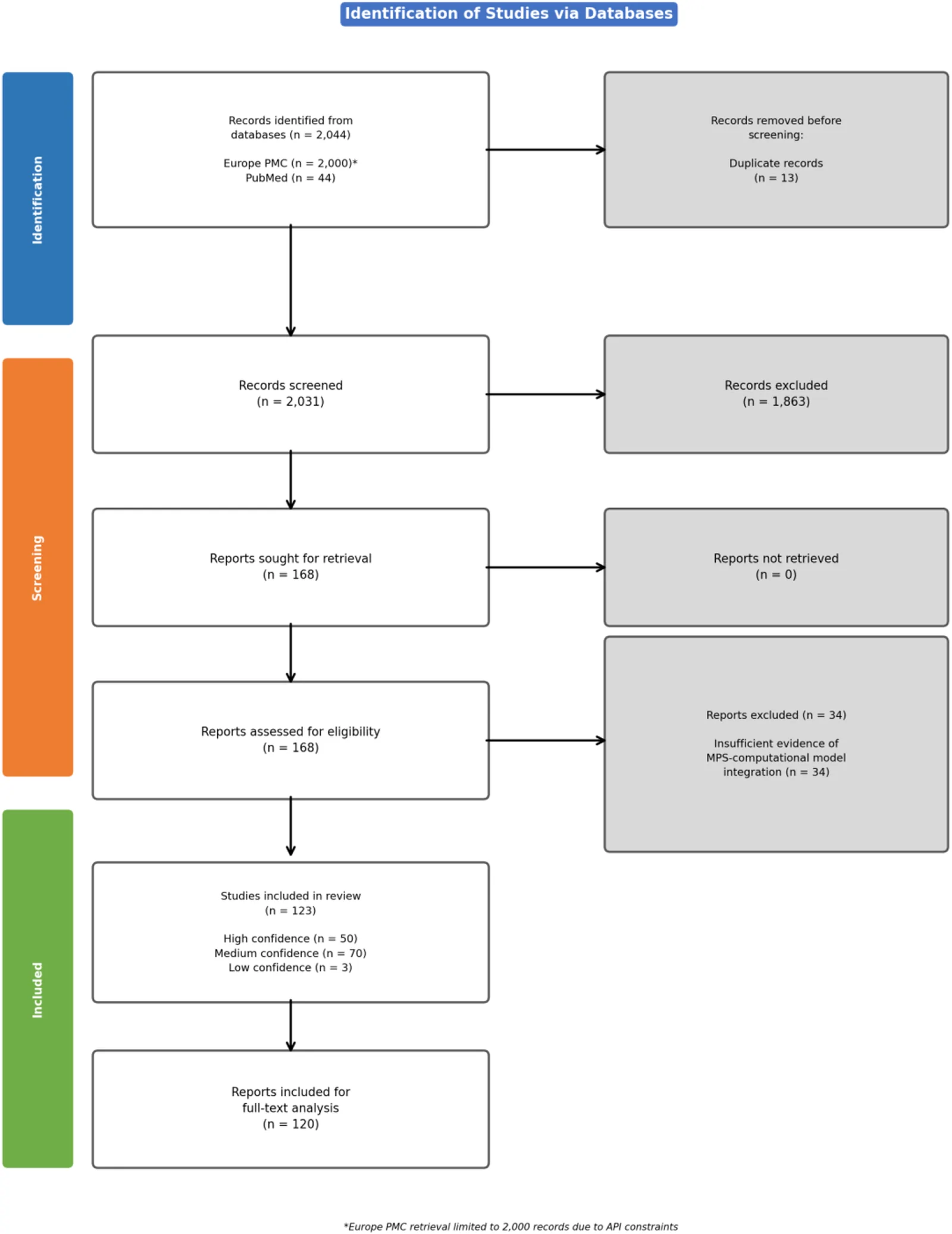
PRISMA Flow Diagram. Flow diagram illustrating the systematic review process from identification through screening to final inclusion. Records were identified from Europe PMC (n = 2,000)* and PubMed (n = 44), yielding a total of 2,044 records. After duplicate removal (n = 13), 2,031 records underwent title/abstract screening. Of these, 1,863 were excluded, leaving 168 for full-text assessment. Following full-text screening, 34 studies were excluded for insufficient evidence of MPS-computational model integration, resulting in 123 studies included in the final synthesis. Of these, 120 studies with accessible full-text PDFs underwent detailed data extraction. Studies were classified by confidence level: high (n = 50), medium (n = 70), and low (n = 3).

The systematic search identified 2,044 records across two databases: 44 from PubMed and 2,000 from Europe PMC (Table 1; Figure 1). The higher yield from Europe PMC reflects its broader full-text indexing and search coverage compared to PubMed’s Title/Abstract-restricted query. After removing 13 duplicate records, 2,031 unique records underwent title and abstract screening. Of these, 1,863 records (91.7%) were excluded for failing to meet the inclusion criteria, primarily because they lacked MPS terminology (n = 1,245), computational modeling terminology (n = 487), or were identified as review articles or excluded publication types (n = 131).

**TABLE 1:** PRISMA flow diagram numbers.

Stage	N
Identification	​
Records from Europe PMC	2,000*
Records from PubMed	44
Total records identified	2,044
Screening	​
Duplicates removed	13
Records screened (title/abstract)	2,031
Records excluded (title/abstract)	1,863
Eligibility	​
Full-text articles assessed	168
Full-text articles excluded	34
Included	​
Studies included in the synthesis	123
Studies with full-text PDF analysis	120

* Europe PMC, retrieval limited to 2,000 records due to API, constraints.

A total of 168 records were assessed for full-text eligibility. After a detailed evaluation, 34 records were excluded due to insufficient evidence of MPS-computational model integration, leaving 123 studies for inclusion in the qualitative synthesis (Figure 1). Full-text PDFs were obtained for 120 studies (97.6%), enabling detailed data extraction and analysis.

### Study characteristics

The characteristics of the 123 included studies are summarized in Table 2. Studies were classified by confidence level: 50 (40.7%) were rated high confidence, 70 (56.9%) were rated medium confidence, and 3 (2.4%) were rated low confidence. The majority of included studies (69.9%) were sourced from Europe PMC, with the remainder (30.1%) from PubMed.

**TABLE 2:** Characteristics of included studies (N = 123).

Characteristic	n	%
Total studies included	123	100.0
Publication year	​	​
2013–2017	11	8.9
2018–2020	21	17.1
2021–2023	44	35.8
2024–2025	47	38.2
Confidence level	​	​
High confidence	50	40.7
Medium confidence	70	56.9
Low confidence	3	2.4
Source database	​	​
Europe PMC	86	69.9
PubMed	37	30.1
Organ systems (studies may include multiple)	​	​
Liver	38	30.9
Vasculature	24	19.5
Gut/Intestine	23	18.7
Immune	21	17.1
Tumor/Cancer	13	10.6
Kidney	13	10.6
Brain	13	10.6
Model types (studies may include multiple)	​	​
PBPK	23	18.7
PK	15	12.2
IVIVE	15	12.2
ML/AI	12	9.8
PK/PD	9	7.3
QSP	6	4.9

Abbreviations: PBPK, physiologically-based pharmacokinetic; PK, pharmacokinetic; IVIVE, in vitro-to-in vivo extrapolation; ML, machine learning; AI, artificial intelligence; PK/PD, pharmacokinetic/pharmacodynamic; QSP, quantitative systems pharmacology.

### Publication trends

**FIGURE 2:**
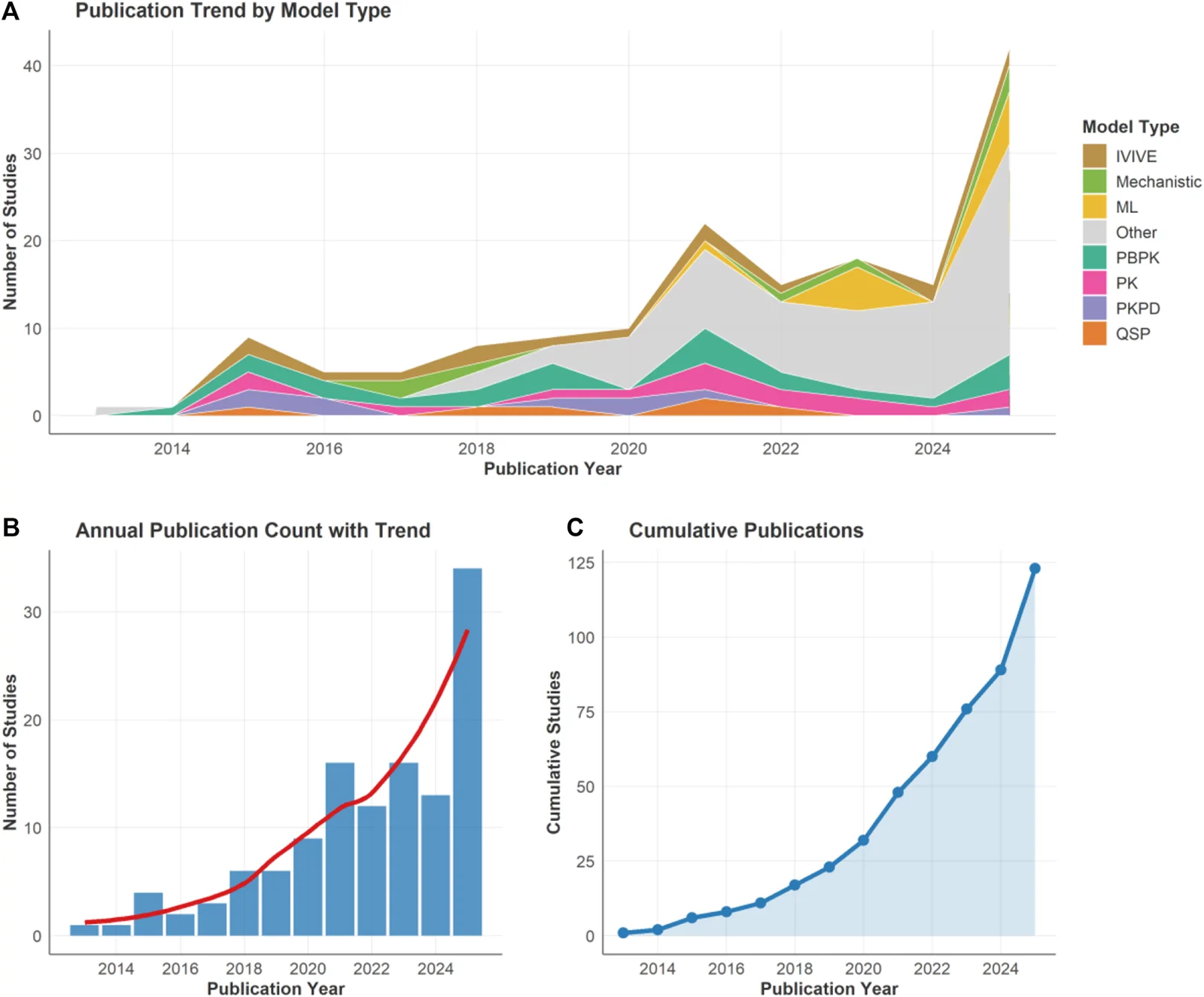
Publication Trends in MPS-Digital Twin Research. **(A)** Stacked area chart showing annual publication counts, stratified by computational model type (PBPK, QSP, PK/PD, PK, Mechanistic, ML, IVIVE, Other), from 2013 to 2025. **(B)** Bar chart showing annual publication counts with an exponential trendline overlay, indicating accelerating growth. **(C)** Cumulative publication plot showing the total number of studies over time, highlighting an exponential increase, particularly after 2020. N = 123 studies.

Publication activity in MPS-digital twin integration research has grown exponentially over the study period (Figure 2). The earliest included studies date to 2013–2014, with only two publications in that period. Growth remained modest through 2017 (cumulative n = 11), accelerated from 2018 to 2020 (n = 21 additional studies), and increased substantially from 2021 to 2023 (n = 44 additional studies). The most recent period (2024–2025) has the highest publication rate, with 47 studies (38.2% of all included studies), including 34 in 2025 alone. This represents a compound annual growth rate (CAGR) of approximately 35% over the study period.

Analysis of publication trends by model type reveals evolving methodological preferences (Figure 2A). PBPK modeling has been consistently represented throughout the study period. IVIVE approaches have seen increasing adoption since 2018. Machine learning (ML) and artificial intelligence approaches emerged more recently, with most ML-integrated studies published after 2021. QSP models, while less frequent overall, have remained present, particularly in studies addressing complex multi-organ interactions.

### Organ systems represented

**FIGURE 3:**
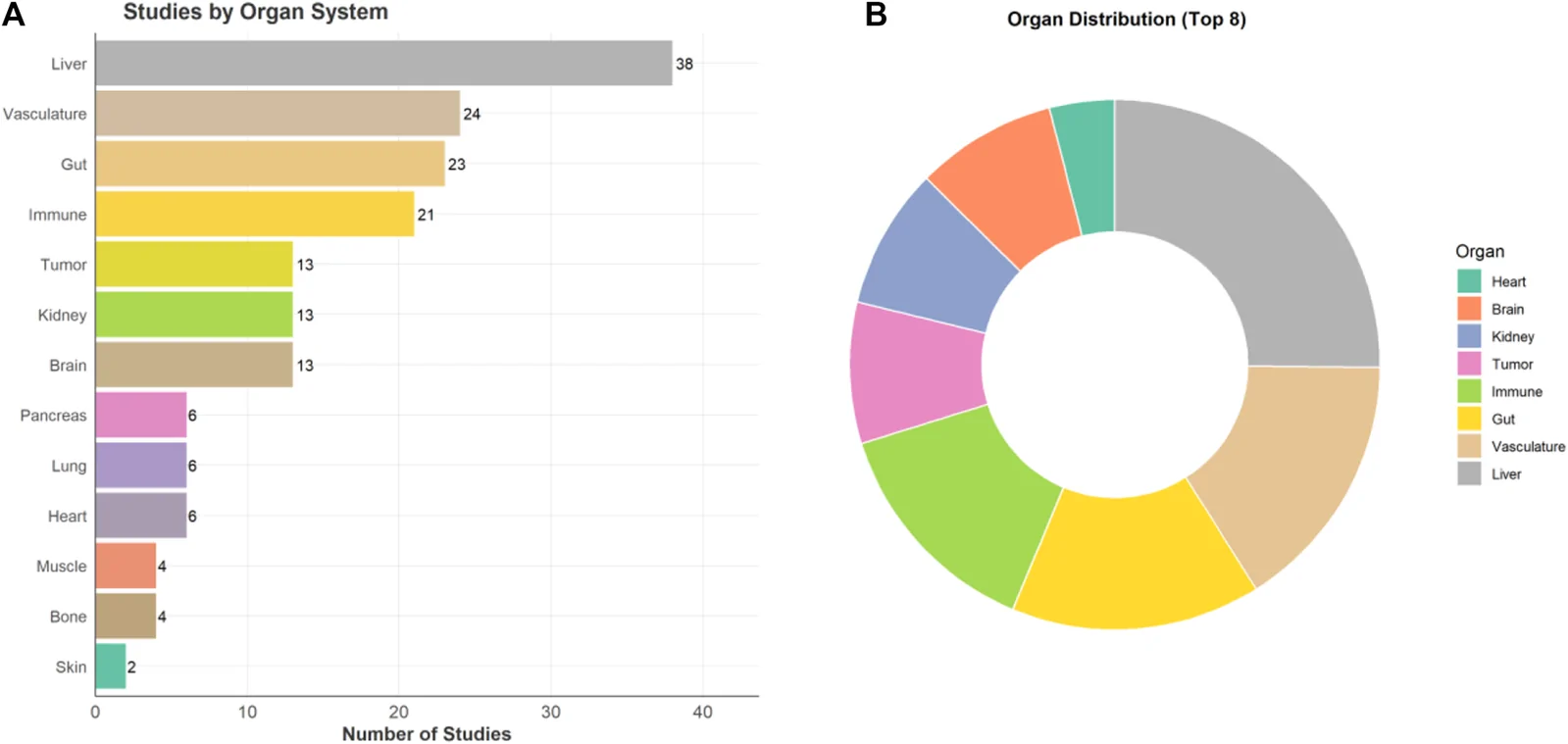
Distribution of Organ Systems in Included Studies. **(A)** Horizontal bar chart showing the number of studies addressing each organ system. The liver is the most represented (n = 38), followed by vasculature (n = 24), gut (n = 23), immune (n = 21), tumor (n = 13), kidney (n = 13), and brain (n = 13). Less-represented organs include the pancreas, lungs, heart, muscle, bone, and skin. **(B)** Donut chart showing the proportional distribution of the top 8 organ systems. Studies may address multiple organ systems.

The distribution of organ systems across the included studies is shown in Figure 3. The liver was the most frequently represented organ system, appearing in 38 studies (30.9%), reflecting the central role of hepatic metabolism in pharmacokinetics and the maturity of liver-on-chip technology. Vasculature/endothelium was represented in 24 studies (19.5%), the gut/intestine in 23 studies (18.7%), and immune system components in 21 studies (17.1%). Tumor/cancer models appeared in 13 studies (10.6%), equal to the number of kidney and brain representations.

Less frequently represented organ systems included the pancreas (n = 6, 4.9%), the lung (n = 6, 4.9%), the heart/cardiac (n = 6, 4.9%), the muscle (n = 4, 3.3%), the bone (n = 4, 3.3%), and the skin (n = 2, 1.6%). Notably, 35 studies (28.5%) did not specify an organ system, often representing multi-organ or body-on-chip platforms without an organ-specific focus.

### Computational model types

Among studies with identifiable model types, PBPK models were most prevalent (n = 23, 18.7%), followed by pharmacokinetic (PK) models (n = 15, 12.2%), IVIVE approaches (n = 15, 12.2%), machine learning methods (n = 12, 9.8%), PK/PD models (n = 9, 7.3%), mechanistic models (n = 8, 6.5%), and QSP models (n = 6, 4.9%). A substantial proportion of studies (n = 72, 58.5%) did not clearly specify the computational model type in the abstract, though the full text provided more detailed methodological descriptions.

**FIGURE 4:**
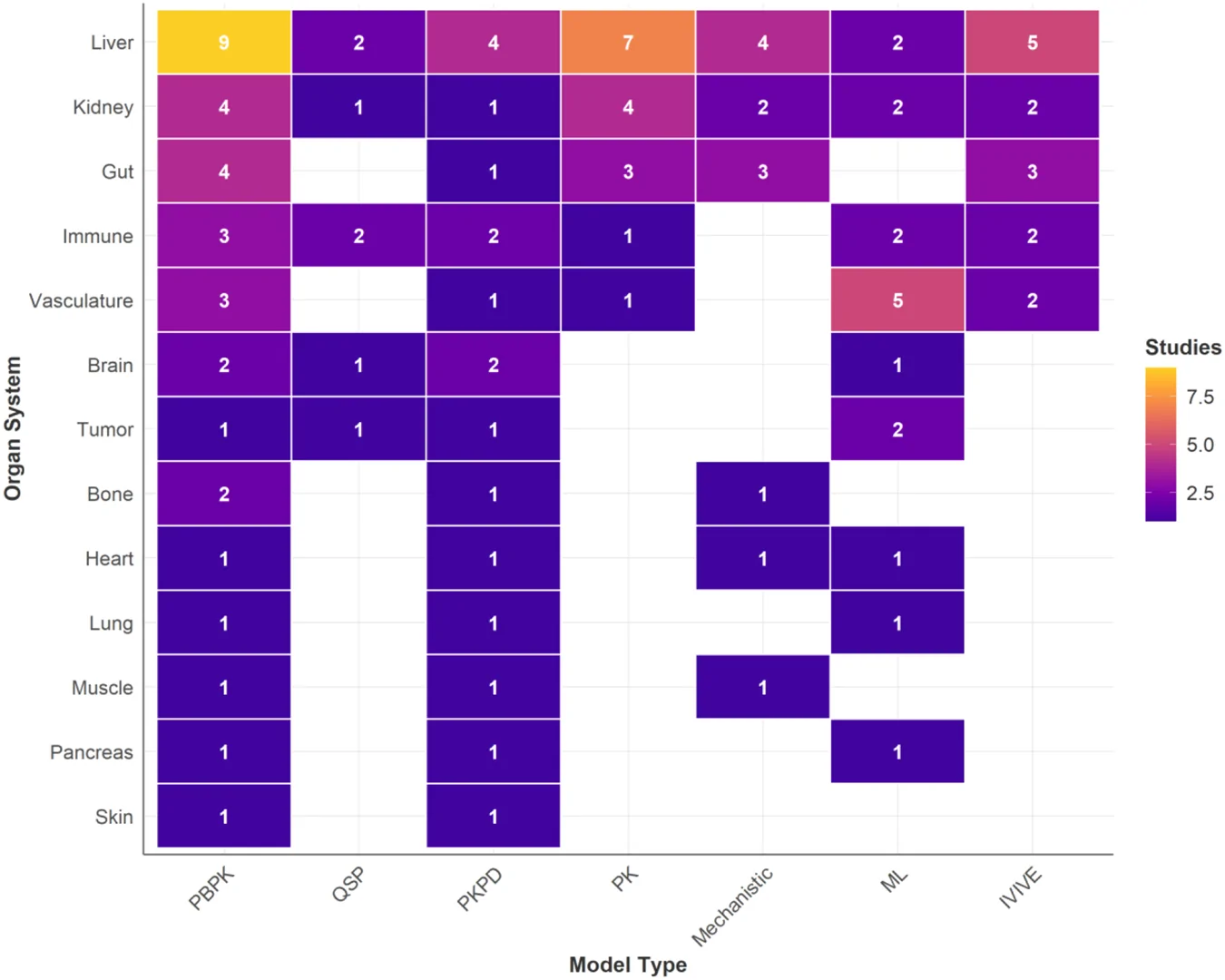
Organ System × Model Type Matrix. Heatmap showing the number of studies for each combination of organ system and computational model type. Color intensity represents study count, with values shown in each cell. The matrix indicates that liver-PBPK (n = 9) and liver-PK (n = 7) are the most established, while many organ-model combinations remain unexplored (represented by empty cells), highlighting research gaps.

The organ-model type matrix (Figure 4) reveals distinct patterns in methodological approaches across organ systems. Liver studies showed the broadest diversity of model types, with strong representation across PBPK (n = 9), PK (n = 7), IVIVE (n = 5), PK/PD (n = 4), and mechanistic models (n = 4). Kidney studies also employed diverse approaches, with notable applications of PBPK (n = 4), PK (n = 4), and IVIVE (n = 2). Studies of vasculature and the immune system showed a preference for ML approaches, possibly reflecting the complex, multi-parametric nature of these systems.

### Application domains

**FIGURE 5:**
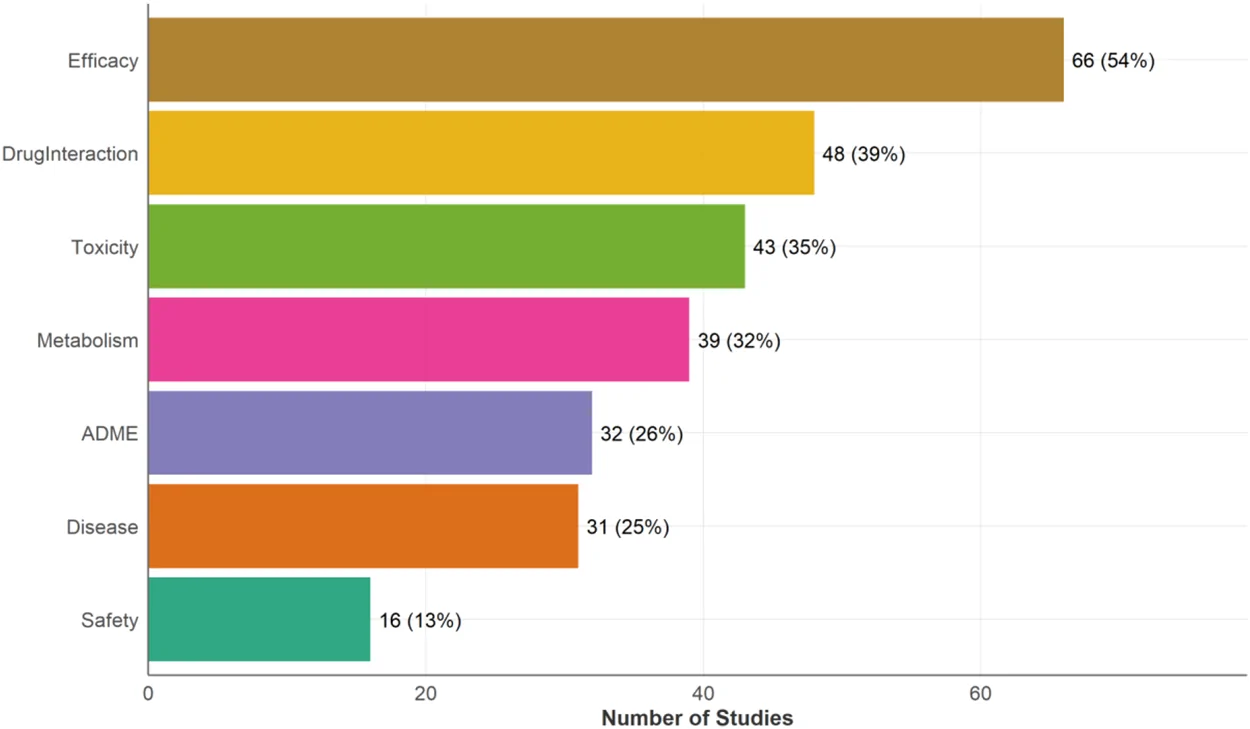
Application Types in Included Studies. Horizontal bar chart showing the number and percentage of studies addressing each application domain. Efficacy assessment is the most common application (n = 66, 54%), followed by drug-drug interactions (n = 48, 39%), toxicity (n = 43, 35%), metabolism (n = 39, 32%), ADME (n = 32, 26%), disease modeling (n = 31, 25%), and safety (n = 16, 13%). Studies may address multiple application domains. N = 123 studies.

Studies were categorized by their primary application domains (Figure 5). Efficacy assessment was the most common application, addressed in 66 studies (53.7%), followed by drug-drug interaction (DDI) prediction in 48 studies (39.0%) and toxicity assessment in 43 studies (35.0%). Metabolism studies accounted for 39 studies (31.7%), ADME characterization for 32 (26.0%), disease modeling for 31 (25.2%), and safety assessment for 16 (13.0%). Studies frequently addressed multiple application domains, reflecting the versatility of integrated MPS-computational approaches.

### Research gap analysis

A systematic gap analysis was conducted to identify underexplored organ-model combinations (Supplementary Figure S3). Research areas were classified as “Established” (>5 studies), “Developing” (3-5 studies), “Emerging” (1-2 studies), or “Gap” (0 studies). The analysis identified liver-PBPK and liver-PK as the only established combinations. Developing areas included kidney-PBPK, gut-PBPK, gut-PK, vasculature-ML, and immune-PBPK.

Significant research gaps were identified across multiple organ-model combinations, particularly for less-studied organs (fat, bone, muscle, skin, pancreas) across all model types and for emerging model types (mechanistic models, QSP) across most organ systems. The gap analysis highlights opportunities for future research to expand the evidence base for underexplored combinations.

### Software and computational tools

**FIGURE 6:**
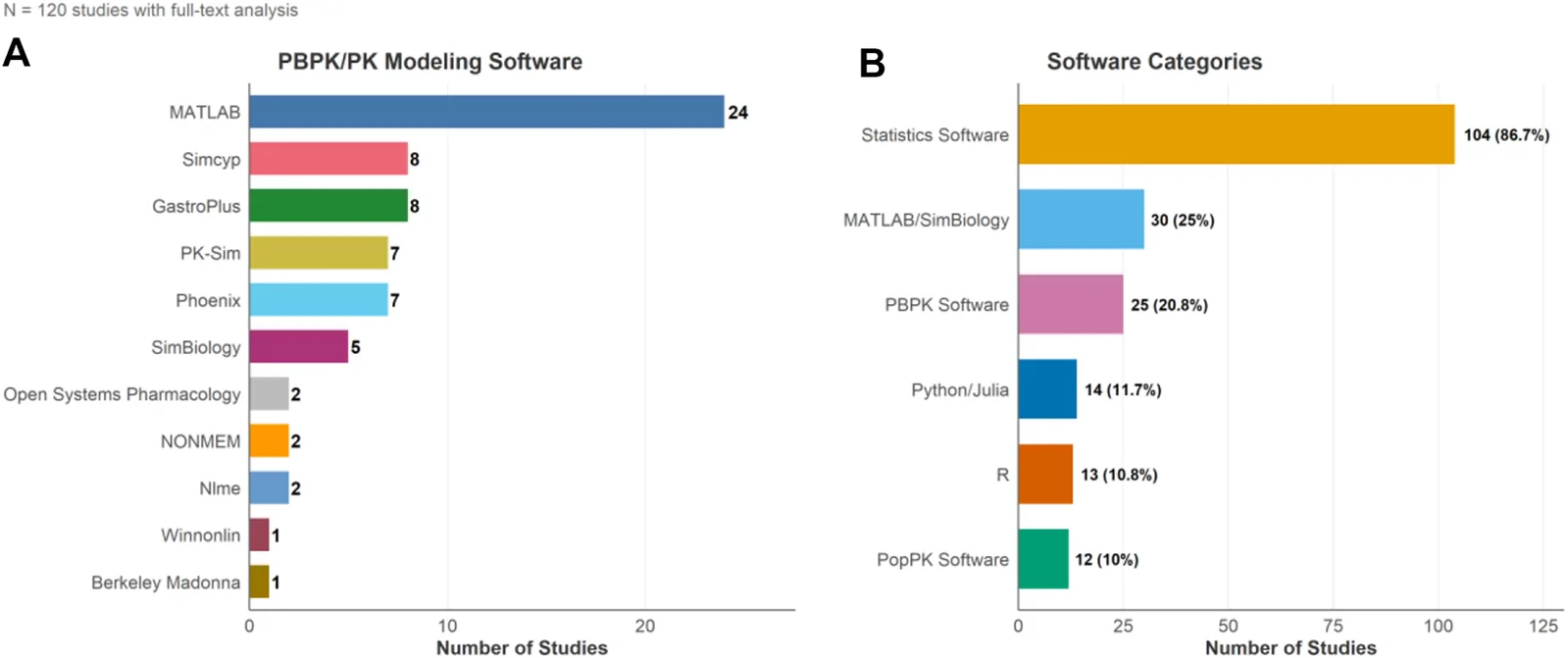
Software Tools Used in MPS-Digital Twin Integration Studies. **(A)** Bar chart showing individual PBPK/PK modeling software usage, with MATLAB most prevalent (n = 24), followed by Simcyp (n = 8), GastroPlus (n = 8), PK-Sim (n = 7), Phoenix (n = 7), and SimBiology (n = 5). **(B)** Software category distribution, with Statistics Software (n = 104, 86.7%), MATLAB/SimBiology (n = 30, 25%), PBPK Software (n = 25, 20.8%), Python/Julia (n = 14, 11.7%), R (n = 13, 10.8%), and PopPK Software (n = 12, 10%). N = 120 studies with full-text analysis.

Full-text analysis of 120 studies identified the computational software and tools used (Figure 6). Among PBPK/PK modeling software, MATLAB was most frequently reported (n = 24 studies, 20.0%), followed by Simcyp (n = 8, 6.7%), GastroPlus (n = 8, 6.7%), PK-Sim/Open Systems Pharmacology (n = 7, 5.8%), Phoenix WinNonlin (n = 7, 5.8%), and SimBiology (n = 5, 4.2%). Population PK software, including NONMEM (n = 2) and Monolix (n = 2), showed limited adoption.

Analysis by software category showed that statistical software (primarily GraphPad Prism and SAS) was most prevalent, reported in 104 studies (86.7%), reflecting the need for basic statistical analysis across all studies. MATLAB/SimBiology (25.0%), dedicated PBPK software (20.8%), Python/Julia (11.7%), R (10.8%), and population PK software (10.0%) were used in 30, 25, 14, 13, and 12 studies, respectively. The complete list of software tools identified is provided in Supplementary Table S1.

### MPS platform utilization

**FIGURE 7:**
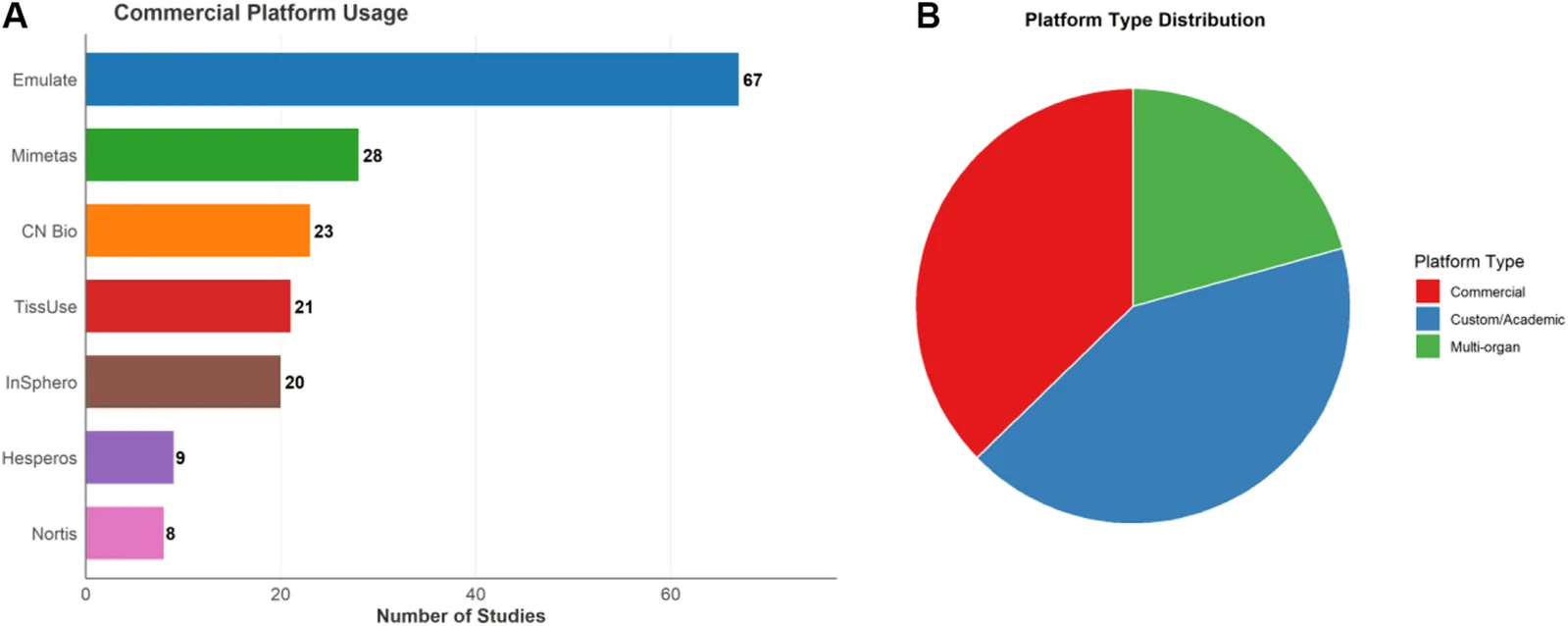
MPS Platform Usage in Digital Twin Studies. **(A)** Bar chart showing commercial platform usage, with Emulate leading (n = 67), followed by Mimetas (n = 28), CN Bio (n = 23), TissUse (n = 21), InSphero (n = 20), Hesperos (n = 9), and Nortis (n = 8). **(B)** Pie chart showing platform type distribution: Custom/Academic platforms are the largest category, followed by Commercial platforms and multi-organ configurations. N = 120 studies with full-text analysis.

The commercial and custom MPS platforms identified in the full-text analysis are shown in Figure 7. Among commercial platforms, Emulate Organ-Chips were most frequently cited (n = 67 mentions across studies), followed by Mimetas OrganoPlate (n = 28), CN Bio PhysioMimix (n = 23), TissUse HUMIMIC (n = 21), InSphero Akura (n = 20), Hesperos systems (n = 9), and Nortis platforms (n = 8). Custom/academic platforms developed in-house were described in 89 studies, the largest category, reflecting continued innovation in MPS design.

Analysis of platform characteristics revealed that microfluidic systems were described in 92 studies (76.7%), with PDMS (polydimethylsiloxane) as the primary fabrication material in 61 studies (50.8%). Multi-organ configurations were explicitly described in 58 studies (48.3%), and pumpless, gravity-driven systems were used in 23 studies (19.2%). A comprehensive summary of the identified commercial MPS platforms is provided in Supplementary Table S2.

### Validation approaches and metrics

**FIGURE 8:**
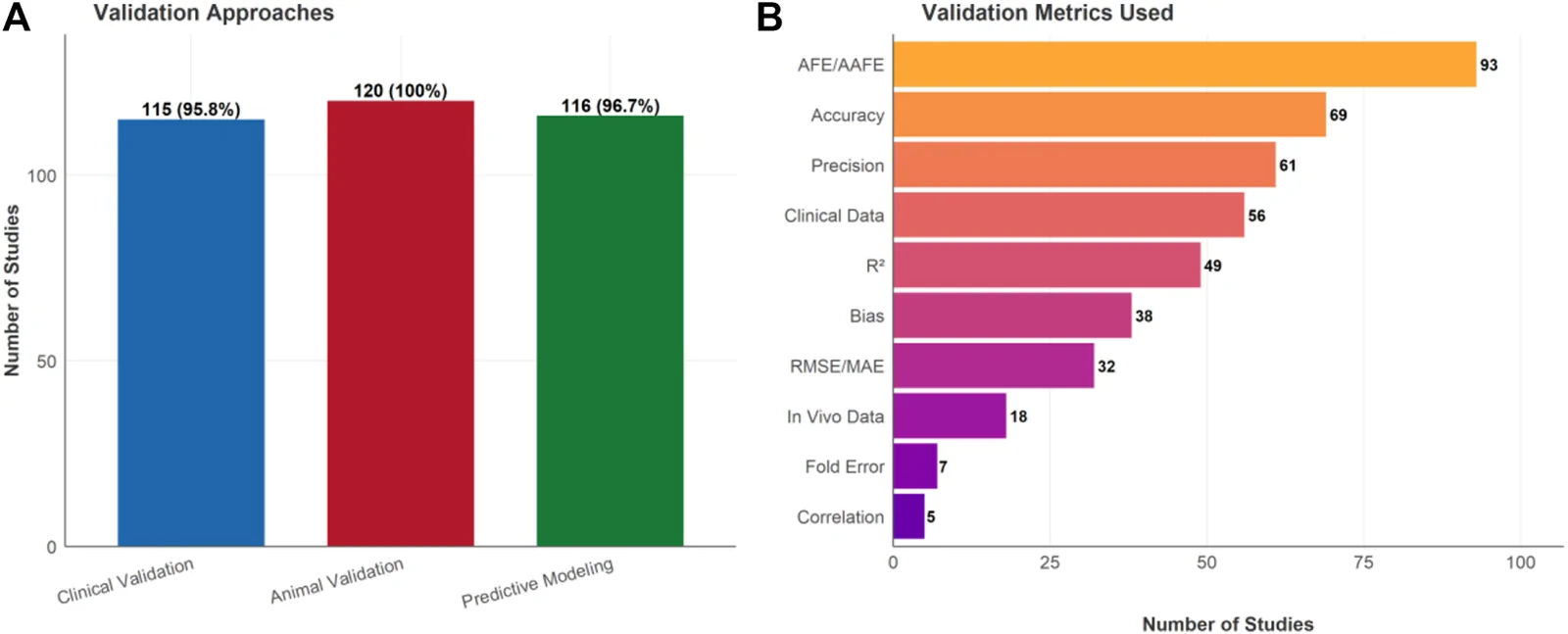
Validation Approaches in MPS-Digital Twin Studies. **(A)** Bar chart showing validation approach terminology detected in full-text analysis: Animal/*in vivo* (n = 120, 100%), Predictive modeling (n = 116, 96.7%), and Clinical/human (n = 115, 95.8%). **(B)** Bar chart displaying validation metrics reported: AFE/AAFE (n = 93), Accuracy (n = 69), Precision (n = 61), Clinical Data (n = 56), R^2^ (n = 49), Bias (n = 38), RMSE/MAE (n = 32), *In Vivo* Data (n = 18), Fold Error (n = 7), and Correlation (n = 5). N = 120 studies.

Validation approaches discussed in the included studies are summarized in Figure 8. Full-text analysis identified validation-related terminology across three categories: animal/*in vivo* comparisons were mentioned in 120 studies (100%), clinical/human data comparisons in 115 studies (95.8%), and predictive modeling approaches in 116 studies (96.7%). These high frequencies reflect the pervasive discussion of validation concepts in MPS-digital twin literature, though the depth and rigor of validation varied considerably across studies.

Among studies reporting specific validation metrics, average fold error (AFE/AAFE) was most prevalent (n = 93, 77.5%), followed by accuracy assessments (n = 69, 57.5%), precision metrics (n = 61, 50.8%), direct comparison with clinical data (n = 56, 46.7%), coefficient of determination (R^2^; n = 49, 40.8%), bias assessment (n = 38, 31.7%), RMSE/MAE (n = 32, 26.7%), and comparison with *in vivo* data (n = 18, 15.0%).

### Pharmacokinetic parameter terminology detected in full-text analysis

**FIGURE 9:**
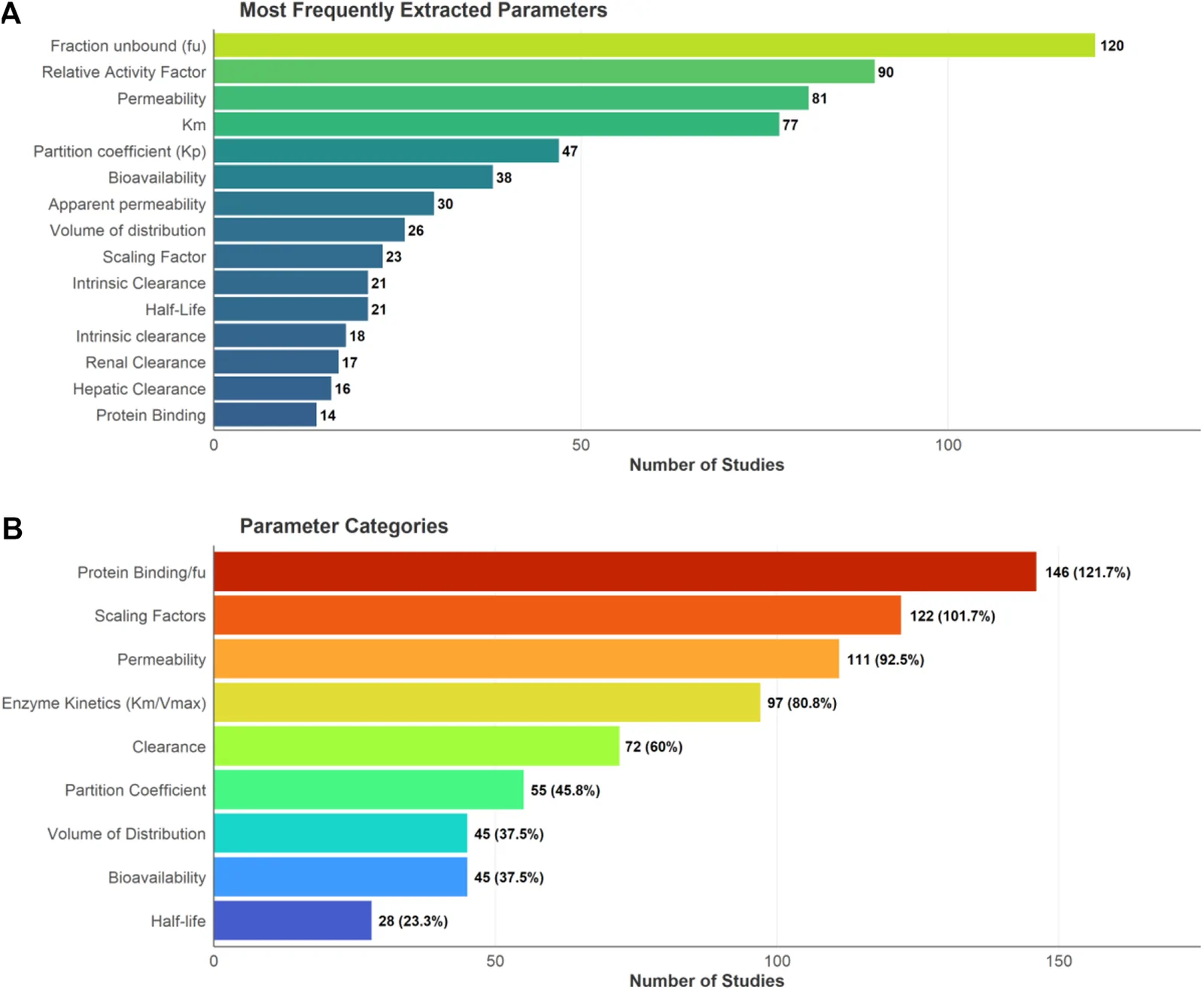
Pharmacokinetic Parameter Terminology Detected in Full-Text Analysis. **(A)** Bar chart showing pharmacokinetic parameter terminology detected in full-text analysis: Fraction unbound (n = 120), Relative Activity Factor (n = 90), permeability (n = 81), Km (n = 77), Partition coefficient (n = 47), Bioavailability (n = 38), Apparent permeability (n = 30), Volume of distribution (n = 26), Scaling factor (n = 23), Intrinsic Clearance (n = 21). **(B)** Bar chart showing parameter categories: Protein Binding/fu (146 mentions, 121.7%), Scaling Factors (122, 101.7%), permeability (111, 92.5%), Enzyme Kinetics (97, 80.8%), Clearance (72, 60.0%). Percentages exceed 100% because multiple parameters are included in each study.

Pharmacokinetic parameters discussed in the context of MPS-to-model integration are shown in Figure 9. Text-mining analysis identified parameter-related terminology across full-text documents. Protein binding and fraction unbound (fu) terminology appeared in 120 studies (100%), reflecting the centrality of this parameter in pharmacokinetic modeling and IVIVE workflows. Scaling factors, including the relative activity factor (RAF), were mentioned in 90 studies (75.0%), permeability-related terms in 81 studies (67.5%), and Michaelis-Menten kinetic parameters (Km) in 77 studies (64.2%). Additional parameters discussed included the partition coefficient (Kp) in 47 studies (39.2%), bioavailability in 38 studies (31.7%), apparent permeability (Papp) in 30 studies (25.0%), volume of distribution (Vd) in 26 studies (21.7%), and intrinsic clearance (CLint) in 21 studies (17.5%). Parameter category analysis revealed that protein binding/fu terms were most prevalent (146 mentions), followed by scaling factors (122 mentions), permeability (111 mentions), enzyme kinetics (97 mentions), and clearance parameters (72 mentions). These frequencies represent terminology detection rather than original experimental derivation, as many studies referenced established parameter values from the literature. In particular, parameters such as fraction unbound are typically obtained from the literature or independent experimental measurements and applied as correction factors within IVIVE and clearance-prediction workflows; their high frequency of detection therefore reflects the centrality of these parameters to the discussion rather than their derivation within the MPS itself.

### Evolution of single-organ vs. multi-organ systems

**FIGURE 10:**
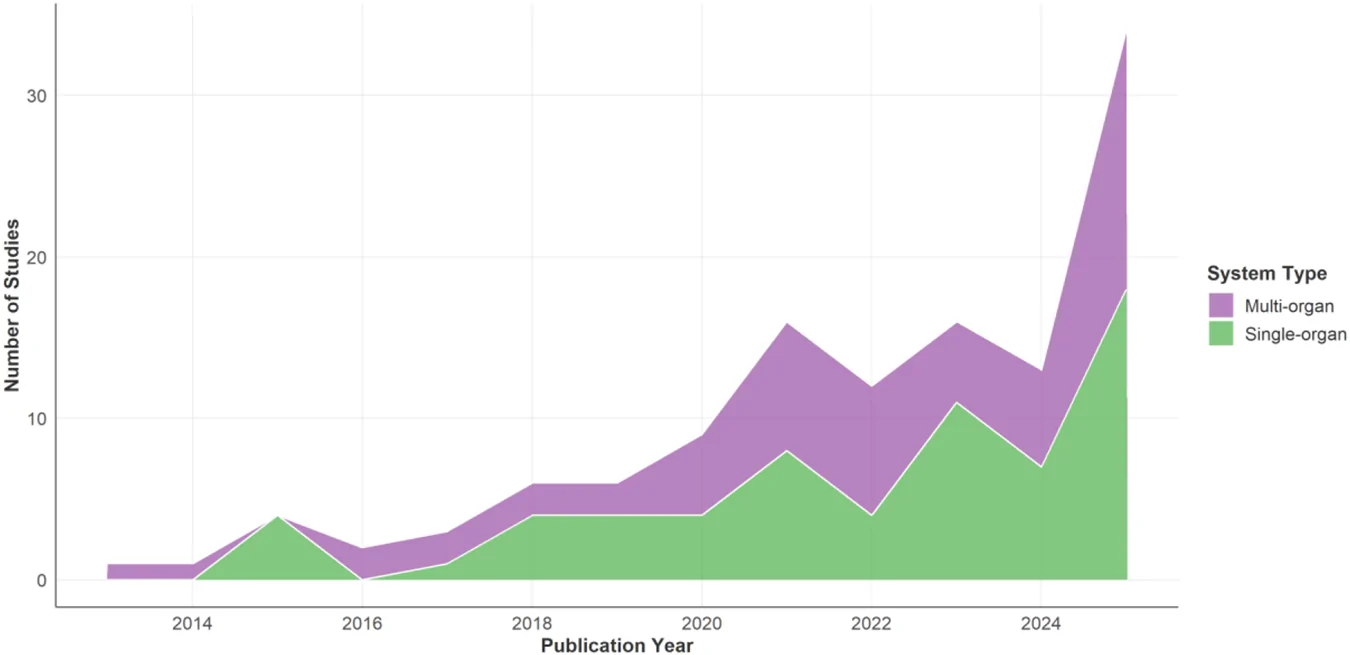
Evolution of Single-organ vs. Multi-organ MPS in Digital Twin Studies. Stacked area chart showing the temporal distribution of single-organ (green) and multi-organ (purple) MPS configurations from 2013 to 2025. Multi-organ systems have seen increasing adoption in recent years, with robust growth after 2021. The trend reflects growing recognition of the importance of organ-organ interactions for comprehensive pharmacokinetic modeling.

Temporal analysis of single-organ versus multi-organ MPS configurations revealed evolving trends (Figure 10). Early studies (2013–2017) predominantly featured single-organ systems, with multi-organ configurations representing a minority. From 2018 onward, multi-organ systems gained increasing adoption, with a notable acceleration after 2021. By 2024–2025, multi-organ systems represented a substantial proportion of published studies, reflecting growing recognition of the importance of organ-organ interactions in pharmacokinetics and pharmacodynamics.

### MPS-digital twin integration framework

**FIGURE 11:**
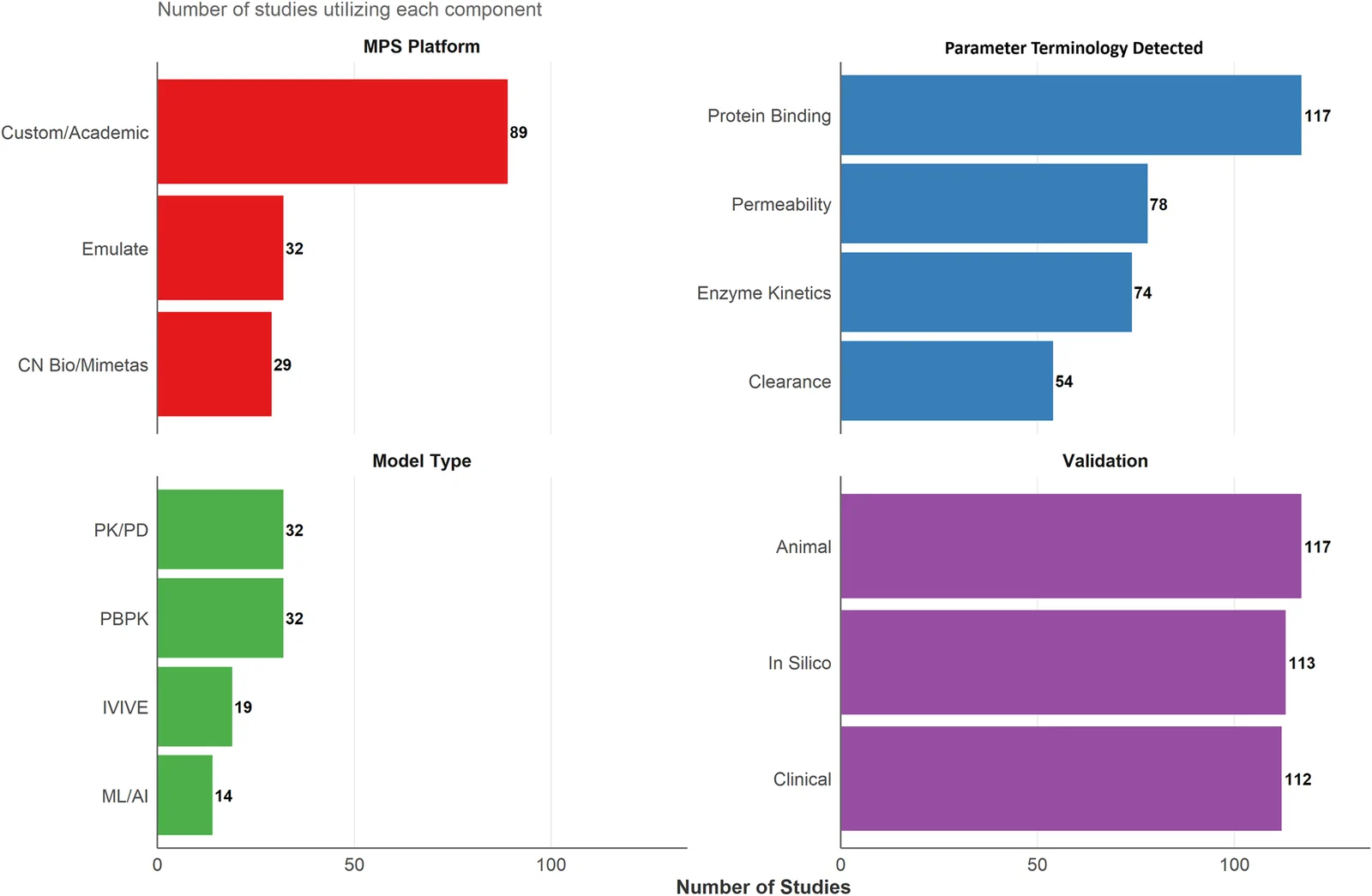
MPS-to-Digital Twin Integration Framework Components. Four-panel summary of study distribution across key integration framework components. MPS Platform: Custom/Academic (n = 89), Emulate (n = 32), CN Bio/Mimetas (n = 29). Parameter Terminology Detected: Protein Binding (n = 117), Permeability (n = 78), Enzyme Kinetics (n = 74), Clearance (n = 54). Model Type: PK/PD (n = 32), PBPK (n = 32), IVIVE (n = 19), ML/AI (n = 14). Validation: Animal (n = 117), In Silico (n = 113), Clinical (n = 112).

A synthesis of integration framework components across studies is presented in Figure 11. The framework encompasses four key domains: MPS platform selection, data extraction, model type selection, and validation approach. For MPS platforms, custom/academic systems predominated (n = 89), followed by Emulate (n = 32) and CN Bio/Mimetas platforms (n = 29). For data extraction, protein binding parameters were most commonly derived (n = 117), followed by permeability (n = 78), enzyme kinetics (n = 74), and clearance values (n = 54).

Model type distribution showed PK/PD models (n = 32) and PBPK models (n = 32) as co-dominant approaches, followed by IVIVE (n = 19) and ML/AI (n = 14). Validation approaches included animal data comparison (n = 117), *in silico* validation (n = 113), and clinical data comparison (n = 112), demonstrating a comprehensive set of validation strategies across the literature. Notably, *in silico* validation was reported in 113 studies (91.9%), indicating that direct comparison of MPS-derived or model outputs against computational (*in silico*) predictions, including PBPK and related approaches, is common across the corpus.

To address the heterogeneity of the machine learning literature, we manually reviewed the twelve studies in which machine learning or artificial intelligence methods were identified (the framework-level tally in Figure 11 reflects automated text detection and includes a small number of records in which these terms appeared only incidentally). Of these twelve studies, only one applied a predictive machine learning model trained on MPS-derived data, namely a neural network predicting oxygen transport from vascularized MPS morphology [48]. Three studies used machine learning as an image-analysis tool applied to MPS readouts, including computer-vision tracking of leukocyte trafficking [49], deep-learning Cell Painting scoring [50], and morphological deep learning for neurotoxicity assessment [51]. Six studies were review or perspective articles that discussed machine learning rather than applying it to MPS data, one study used *in silico* and omics analysis for biomarker identification without a trained predictive model, and one study concerned an unrelated computational application. This distribution indicates that, within the present corpus, machine learning is rarely used as a predictive model trained on MPS data and more often serves an image-analysis or discussion role. A study-level classification is provided in Supplementary Table S3.

**FIGURE 12:**
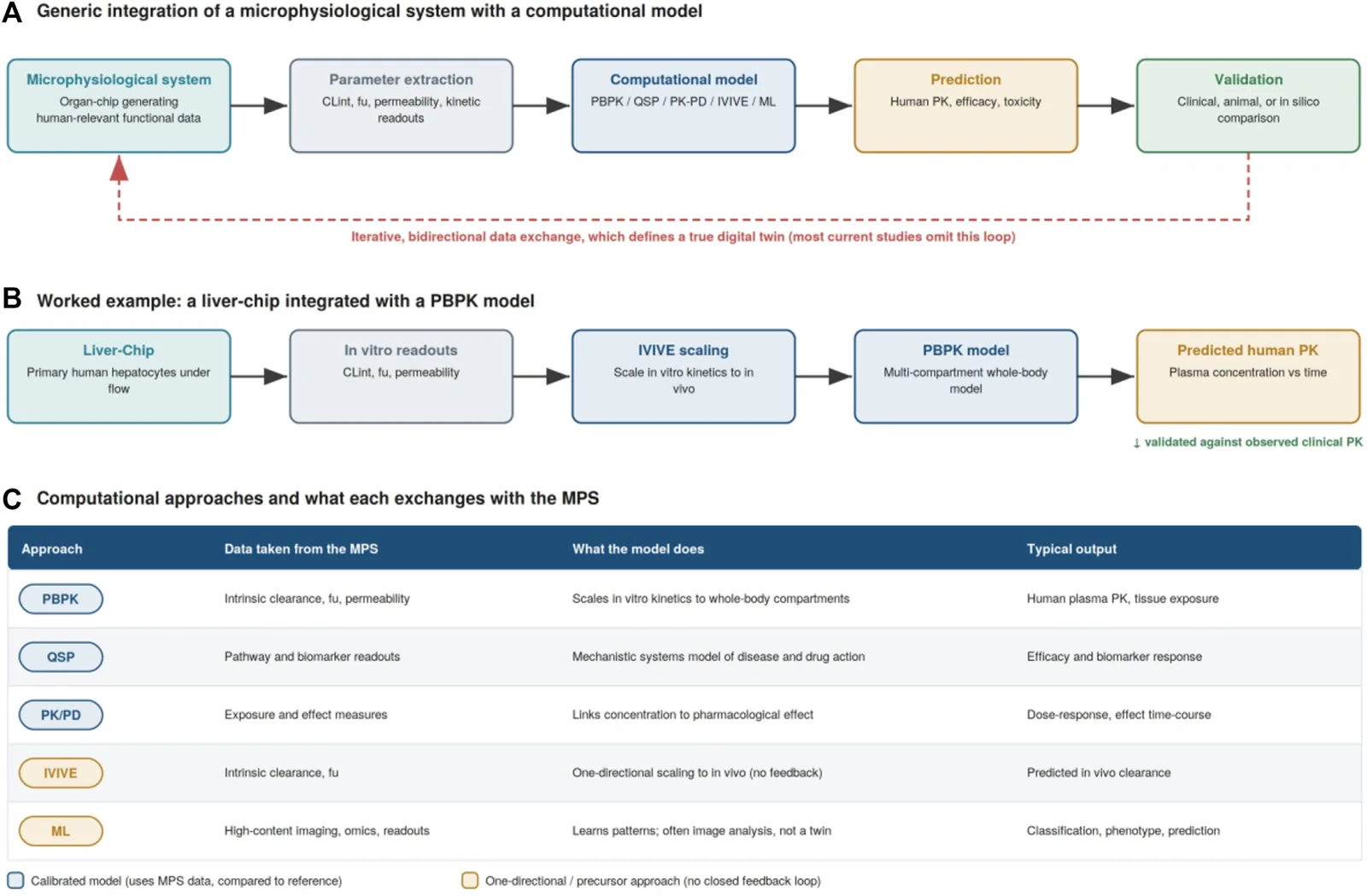
Integration of microphysiological systems with computational and digital twin modeling. **(A)** Generic workflow in which an organ-chip generates human-relevant data, parameters are extracted, a computational model produces predictions, and predictions are validated; the dashed loop denotes the iterative, bidirectional data exchange that defines a true digital twin and that most current studies do not implement. **(B)** Worked example of a liver-chip integrated with a physiologically based pharmacokinetic (PBPK) model, in which *in vitro* readouts (intrinsic clearance, fraction unbound, permeability) are scaled by *in vitro* to *in vivo* extrapolation (IVIVE) into a multi-compartment model to predict human plasma pharmacokinetics, validated against observed clinical data. **(C)** Principal computational approaches, the data each draws from the MPS, the modeling step performed, and the typical output; PBPK, QSP, and PK/PD are calibrated models, whereas IVIVE and many machine learning (ML) applications are one-directional precursor approaches lacking a closed feedback loop.

To clarify how these approaches integrate with MPS in practice, Figure 12 presents the generic integration workflow, a worked liver-chip-to-PBPK example, and a comparison of the data exchanged and outputs produced by each computational approach.

To illustrate how these approaches operate in practice, Table 3 presents one representative study for each computational approach, summarizing how the model is integrated with the MPS and the principal finding of the study.

**TABLE 3:** Representative studies illustrating the integration of each computational approach with a microphysiological system.

Approach	Representative study	How the integration works	Key finding
PBPK	Tsamandouras et al., 2017 [52]	Metabolic depletion of six drugs was measured in a perfused three-dimensional human liver MPS using hepatocytes from five donors, and the *in vitro* clearance data were integrated into a population physiologically based pharmacokinetic model for *in vitro* to *in vivo* translation	Predicted clearances correlated with observed *in vivo* values, and the population model reproduced clinical concentration-time profiles and their variability for lidocaine, the first demonstration of population variability in drug metabolism within an MPS.
QSP	Maass et al., 2019 [53]	Drug-induced proximal tubule injury was quantified in a kidney MPS using the biomarker KIM-1, and a quantitative systems pharmacology model used these readouts for *in vitro* to *in vivo* translation	The model translated MPS injury readouts to the clinical setting and identified favourable dosing regimens for a nephrotoxic drug, supporting preclinical nephrotoxicity prediction
**PK/PD**	Shinha et al., 2020 [54]	A multi-organ-on-a-chip linked a liver (metabolic) compartment to a cancer (target) compartment, and a PK-PD model described kinetics of the prodrug CPT-11 and its metabolite across the chip, including inhibition by simvastatin and ritonavir	PK-PD model predictions matched the chip co-administration experiments, demonstrating that organ-on-chip combined with PK-PD modelling can predict drug-drug interactions
IVIVE	Sakolish et al., 2021 [55]	Hepatic clearance of seven compounds was measured in a four-cell liver acinus MPS using primary or iPSC-derived hepatocytes, then scaled to *in vivo* clearance by *in vitro* to *in vivo* extrapolation and compared with clinical values	iPSC-hepatocyte MPS gave more precise clearance estimates than suspension or 2D cultures but, like other MPS, underestimated *in vivo* clearance, indicating that an empirical scaling factor is required
ML	Tronolone et al., 2023 [48]	A chained neural network was trained on morphological metrics from diverse vascularized MPS to output a single vascular network quality index (VNQI)	VNQI correlated with measured oxygen levels better than individual morphological metrics and tracked transplant functional outcomes in a vascularized islet-chip, enabling standardization of organ-chips

Abbreviations: IVIVE, *in vitro* to *in vivo* extrapolation; KIM-1, kidney injury molecule-1; ML, machine learning; MPS, microphysiological system; PBPK, physiologically based pharmacokinetic; PK-PD, pharmacokinetic-pharmacodynamic; QSP, quantitative systems pharmacology; VNQI, vascular network quality index.

### Publishing journals

The distribution of included studies across journals is shown in Supplementary Figure S4. Drug Metabolism and Disposition was the leading journal with three publications, followed by PLoS ONE (n = 2), Micromachines (n = 2), Lab on a Chip (n = 2), Integrative Biology (n = 2), and Clinical and Translational Science (n = 2). The remaining studies were distributed across a wide range of journals, reflecting the interdisciplinary nature of MPS-digital twin research spanning pharmacology, bioengineering, toxicology, and computational biology.

### Economic and translational considerations

While economic and translational themes were widely discussed across the included studies, the depth of quantitative analysis remained limited. In this context, “efficiency improvement” denotes qualitative mentions of increased experimental throughput, automation, scalability, faster turnaround, or reduced reagent and resource use, rather than a quantified economic endpoint. Most studies referenced efficiency improvements (92.5%) and animal reduction aligned with 3Rs principles (73.3%) in qualitative terms. Discussion of return on investment and time savings each appeared in 67.5% of studies. However, only 25.8% of studies included specific cost discussions, and just 10.8% cited dollar amounts. No studies performed formal cost-effectiveness or cost-benefit analyses. Temporal trends showed that discussions of efficiency and animal reduction have increased in prevalence since 2018, while cost quantification has remained consistently low. Temporal trends in economic topic coverage across the full study period are presented in Supplementary Figure S1, and a detailed breakdown of economic topic prevalence and coverage depth is shown in Supplementary Figure S2. The most detailed economic analysis to date is that of Ewart et al. [11], who estimated the potential annual value of Liver-Chip adoption at over $3 billion based on performance characteristics. These findings suggest that rigorous health economic evaluation represents a significant gap in the current literature.

## Discussion

### Summary of principal findings

This systematic review provides the first comprehensive synthesis of the literature on integrating microphysiological systems with digital twin computational modeling for pharmaceutical development. Our analysis of 123 studies published between 2013 and 2025 reveals a rapidly maturing field characterized by exponential growth in publications, methodological diversification, and increasing translational sophistication. The findings demonstrate that MPS-digital twin integration has evolved from proof-of-concept demonstrations to a robust research paradigm with established validation practices and growing regulatory acceptance.

Several key findings emerge from this synthesis. First, the field has experienced remarkable growth, with a compound annual growth rate of approximately 35% and 38.2% of all included studies published in 2024–2025 alone. This acceleration reflects both technological maturation and increasing recognition of the translational value of integrated experimental-computational approaches. Second, the liver dominates as the most studied organ system (30.9%), followed by vasculature (19.5%), gut (18.7%), and immune components (17.1%), a distribution that aligns with the pharmacokinetic and toxicological priorities of drug development. Third, PBPK modeling represents the most prevalent computational approach (18.7%), though the field shows increasing methodological diversity, including machine learning (9.8%), QSP (4.9%), and hybrid approaches.

Notably, the review identified strong validation practices across the literature, with 95.8% of studies discussing clinical/human data comparisons and 77.5% reporting quantitative validation metrics such as average fold error. The high prevalence of custom/academic MPS platforms (74.2%) alongside commercial systems from Emulate (55.8%), Mimetas (23.3%), and CN Bio (19.2%) reflects continued innovation in platform design while commercial standardization advances. The evolution from single-organ to multi-organ systems, particularly pronounced after 2021, indicates growing recognition that organ-organ interactions are essential for accurate pharmacokinetic prediction.

### Comparison with existing literature

Our findings align with and extend previous narrative reviews of MPS technology and computational modeling in pharmaceutical development. Ingber’s comprehensive review of organs-on-chips for disease modeling and drug development [17] highlighted the transformative potential of these technologies but did not systematically quantify integration with computational approaches. Similarly, Marx and colleagues' biology-inspired MPS roadmap [20] emphasized the need for standardization and regulatory qualification, but preceded the recent acceleration in MPS-digital twin integration research. Our systematic approach provides the first quantitative characterization of this integration landscape.

The dominance of liver-focused research identified in our review is consistent with the IQ MPS Affiliate’s prioritization of hepatic models for safety risk assessment [39, 41]. Baudy and colleagues' liver MPS development guidelines established qualification frameworks that have guided subsequent research, and our finding that liver-PBPK represents the only truly established organ-model combination (>5 studies) validates this strategic focus [41]. However, our gap analysis reveals that even well-studied combinations, such as liver-PBPK, comprise only 9 studies, indicating substantial opportunities for consolidating evidence.

The validation metrics reported in our review compare favorably with traditional preclinical approaches. Ewart and colleagues demonstrated that Liver-Chip technology achieved 87% sensitivity and 100% specificity for predicting drug-induced liver injury [11], substantially outperforming the 50–60% concordance typically observed with animal models [42, 43]. Our finding that average fold error (AFE/AAFE) is the most commonly reported validation metric (77.5%) reflects adoption of regulatory-accepted standards for PBPK model qualification, suggesting that the field is aligning with expectations for eventual regulatory submission.

### Interpretation of key findings

#### Organ system distribution and research priorities

The concentration of research on liver, gut, and vascular systems reflects rational prioritization based on pharmacokinetic relevance and technological maturity. The liver’s central role in drug metabolism, combined with the clinical and economic burden of drug-induced liver injury, justifies its predominance. Similarly, gut and vascular models address critical determinants of oral bioavailability and systemic distribution. However, the relative underrepresentation of organs such as the lung (4.9%), skin (1.6%), and pancreas (4.9%) represents missed opportunities, particularly given the therapeutic importance of respiratory diseases, dermatological conditions, and metabolic disorders.

The substantial representation of tumor/cancer models (10.6%) and immune system components (17.1%) reflects the field’s expansion beyond traditional pharmacokinetic applications toward efficacy prediction in immuno-oncology. This evolution aligns with the pharmaceutical industry’s strategic shift toward oncology and immunotherapy, where traditional animal models have shown particularly poor predictive validity for human responses.

#### Computational modeling approaches

The predominance of PBPK and PK modeling approaches reflects the mature theoretical frameworks and regulatory acceptance of these methodologies for pharmacokinetic prediction. PBPK models provide mechanistic whole-body representations that naturally accommodate MPS-derived parameters such as intrinsic clearance, permeability, and protein binding. The emergence of machine learning approaches (9.8%), particularly after 2021, represents an important methodological evolution that may address complex, high-dimensional datasets where mechanistic understanding remains incomplete.

The relatively limited adoption of QSP approaches (4.9%) is noteworthy given their potential for efficacy prediction and disease modeling. This may reflect the greater complexity of QSP model development, the need for extensive biological knowledge, and the longer timelines required for model qualification. As MPS platforms increasingly incorporate disease-relevant phenotypes and multi-organ interactions, QSP integration may become more prevalent.

#### Platform landscape and standardization

The coexistence of custom/academic platforms (74.2%) and commercial systems reflects the field’s developmental stage. Custom platforms enable continued innovation in design, cell sourcing, and application-specific optimization, while commercial platforms offer standardization, reproducibility, and scalability essential for pharmaceutical adoption. Emulate’s market leadership (55.8%) likely reflects their early mover advantage, regulatory engagement through the ISTAND program, and the foundational scientific contributions from Ingber and colleagues [17, 22, 40].

The diversity of commercial platforms, including Mimetas OrganoPlate, CN Bio PhysioMimix, TissUse HUMIMIC, and InSphero Akura, provides pharmaceutical end-users with options suited to different applications and throughput requirements. However, this diversity also presents challenges for cross-platform comparison and method standardization. The IQ MPS Affiliate’s ongoing efforts to establish qualification guidelines and performance benchmarks address this need [21, 39, 41].

#### Validation practices and regulatory readiness

The high prevalence of validation against clinical/human data (95.8%) and the widespread use of quantitative metrics such as AFE (77.5%) and R^2^ (40.8%) indicate that the field has adopted rigorous validation standards consistent with regulatory expectations. These practices reflect the influence of PBPK regulatory guidance documents and the IQ consortium’s qualification frameworks. The finding that nearly all studies (100%) discussed comparisons with animal data, while fewer (46.7%) reported direct clinical comparisons, suggests opportunities to strengthen the translational evidence base.

The software landscape, dominated by MATLAB (20.0%) and commercial PBPK platforms (Simcyp 6.7%, GastroPlus 6.7%, PK-Sim 5.8%), reflects the computational infrastructure supporting MPS-digital twin integration. The emergence of Python (10.0%) and R (10.8%) indicates growing adoption of open-source tools that may facilitate reproducibility and method sharing. The limited use of population PK software (NONMEM: 1.7%; Monolix: 1.7%) suggests opportunities to integrate MPS data into clinical pharmacometric workflows.

### Research gaps and future directions

Our systematic gap analysis identified substantial opportunities for research expansion. The organ-model matrix revealed that only liver-PBPK and liver-PK qualify as established combinations (>5 studies), while numerous organ-model pairings remain unexplored or minimally investigated. Priority areas for future research include:

First, expansion of kidney MPS-computational integration beyond current PBPK applications to include mechanistic models of renal drug handling, transporters, and nephrotoxicity prediction.

Second, the development of lung MPS digital twin frameworks for inhaled drug delivery, respiratory disease modeling, and pulmonary toxicity prediction.

Third, integration of brain/blood-brain barrier MPS with computational models for CNS drug development, an area of high unmet medical need with particularly challenging translational barriers.

Fourth, advancement of multi-organ MPS platforms with corresponding multi-compartment computational models that capture organ-organ interactions, enterohepatic recirculation, and systemic pharmacokinetic profiles. The temporal trend toward multi-organ systems identified in our review (accelerating after 2021) should continue with increasing sophistication.

Fifth, integration of QSP approaches with MPS platforms for disease modeling and efficacy prediction, extending beyond the current pharmacokinetic focus toward pharmacodynamic and therapeutic outcome prediction.

Sixth, standardization of MPS-derived parameter extraction methodologies, including harmonized protocols for measuring intrinsic clearance, permeability, and scaling factors.

Seventh, development of uncertainty quantification frameworks that propagate experimental variability from MPS measurements through computational model predictions to clinical exposure estimates.

Eighth, establishment of public databases and data-sharing initiatives that enable meta-analysis of MPS-derived parameters across platforms and laboratories.

To translate these gaps into concrete next steps, we propose the following priorities for the field:

1. Standardize the reporting of MPS-model integration, including the MPS platform, the parameters exchanged, the model type, and the validation strategy, to enable cross-study comparison.
2. Develop genuinely bidirectional, iteratively updated data pipelines that couple MPS measurements with computational models, moving beyond one-directional extrapolation toward true digital twins.
3. Prioritize under-developed organ-model combinations, particularly kidney, lung, and brain, and expand multi-organ systems paired with multi-compartment models.
4. Conduct formal cost-effectiveness and cost-benefit analyses to demonstrate value to pharmaceutical decision-makers, addressing the economic evidence gap identified in this review.
5. Establish shared benchmark datasets and reference cases that allow validation accuracy to be compared across studies and would support future meta-analysis.
6. Pursue regulatory qualification of MPS-model approaches for organ systems beyond the liver, building on the precedent of the FDA ISTAND [40] pathway.

### Regulatory and translational implications

The regulatory landscape for MPS-digital twin integration is evolving rapidly. The FDA Modernization Act 2.0, enacted in December 2022, eliminated the federal mandate requiring animal testing and explicitly recognized organ chips and microphysiological systems as acceptable alternatives [38]. The FDA’s April 2025 Roadmap to Reducing Animal Testing in Preclinical Safety Studies [39] further signals regulatory readiness to accept MPS-derived data in drug development applications. The acceptance of the first Liver-Chip into the FDA’s ISTAND program in September 2024 represents a milestone toward formal qualification.

Our finding that 92.5% of studies discussed efficiency and throughput improvements, while 73.3% addressed animal reduction aligned with 3Rs principles, indicates strong awareness of the translational value proposition [11, 40]. However, as detailed in results, rigorous health economic analyses remain scarce, with most studies limiting their discussion to qualitative claims of efficiency rather than formal cost-effectiveness evaluation.

For adoption in the pharmaceutical industry, our findings suggest that liver-focused applications are most ready for implementation, with established platforms, validation frameworks, and regulatory precedent. Companies considering MPS-digital twin integration should prioritize liver toxicity prediction as an entry point while building capabilities for broader application. The IQ MPS Affiliate’s qualification guidelines [21, 41], provide a roadmap for internal validation and regulatory engagement.

Developments since the search cutoff. Although this review systematically covers literature indexed through 31 December 2025, the field has continued to advance. Notably, the Emulate Liver-Chip S1 has progressed through the FDA ISTAND qualification pathway as the first organ-on-a-chip technology accepted into the program [40], reinforcing the regulatory momentum described above. We note these developments as illustrative context only, as they were not part of the systematically screened corpus. The rapidly evolving nature of the field means the quantitative findings reported here represent a snapshot that will require periodic updating.

### Defining the digital twin: a maturity spectrum

The term “digital twin” is central to this review, yet it is applied inconsistently across the literature, often as a synonym for any patient- or system-specific computational model. As defined in the introduction, a true digital twin is characterized by bidirectional and iteratively updated information flow, in which the virtual model is continuously initialized and refined by measurements from the physical system and, in turn, informs decisions about that system. Judged against this definition, the included studies are better understood as occupying a maturity spectrum rather than a single category. At the first level are one-directional extrapolation approaches, such as IVIVE and scaling methods, in which parameters measured in an MPS are transferred to a model without a closed feedback loop. At the second level are calibrated and parameterized models, including most PBPK, QSP, and PK/PD studies, in which MPS data are used to fit or validate a model that is then compared against clinical or animal data. At the third level are fully bidirectional digital twins, in which the model and the physical MPS are coupled in an iterative, continuously updated cycle. The majority of studies in this corpus fall within the first two levels and are therefore best regarded as precursors to, or components of, digital twins rather than fully realized twins. Consistent with this, manual review of the machine learning studies found that only one applied a predictive model trained on MPS-derived data, while most used machine learning for image analysis of MPS readouts or discussed it in review and perspective articles, confirming that the machine-learning-labelled literature sits largely at the precursor end of this spectrum. Recognizing this distinction clarifies both the present maturity of the field and the methodological developments, particularly closed-loop data integration, that will be required to realize genuine digital twins for pharmaceutical development.

### Limitations

This systematic review has several limitations that should be considered when interpreting the findings. First, the automated screening methodology, while ensuring reproducibility and consistency, may have excluded relevant studies that did not contain the specified terminology in titles or abstracts. Manual screening of borderline cases and expert consultation could have improved sensitivity. Second, the text-mining approach for full-text data extraction captures mentions of terminology rather than verified experimental reporting; consequently, high-frequency parameters such as fraction unbound and validation approaches reflect discussion prevalence rather than universal experimental derivation. Third, the search was restricted to English-language publications, potentially excluding relevant research published in other languages. Fourth, although the search was executed on 14 December 2025 and captured records indexed through 31 December 2025, and thus reflects actual indexed literature rather than projected data, records from the final weeks of 2025 may be incompletely indexed, and the rapidly evolving field means very recent developments may be missed. Fifth, the confidence level classification, while based on predefined criteria, involves subjective judgment regarding evidence strength. Sixth, publication bias may affect the findings, as studies with negative results or failed predictions may be underrepresented in the literature. Seventh, the heterogeneity of included studies, spanning diverse organ systems, model types, applications, and platforms, precluded quantitative meta-analysis of prediction accuracy or other outcome measures. Future reviews focusing on specific organ-model combinations may enable such analyses. Eighth, proprietary pharmaceutical research not published in the peer-reviewed literature is not captured, potentially underestimating industry adoption and application scope. Ninth, the Europe PMC search retrieved a maximum of 2,000 records due to API limitations, potentially missing additional relevant studies indexed in that database. However, the high screening exclusion rate (91.7%) suggests that comprehensive retrieval was achieved for studies meeting the inclusion criteria. Finally, the rapidly evolving nature of both MPS technology and computational modeling means that findings may require periodic updating as the field advances.

### Conclusion

This systematic review provides a comprehensive characterization of the MPS-digital twin integration landscape for pharmaceutical development. Based on an analysis of 123 studies published between 2013 and 2025, we draw the following conclusions:

First, MPS-digital twin integration represents a rapidly maturing field with exponential publication growth (CAGR ∼35%) and increasing methodological sophistication. The concentration of 38.2% of publications in 2024–2025 indicates accelerating research momentum aligned with regulatory and industry interest.

Second, liver-focused applications represent the most mature area, with established validation frameworks, commercial platform availability, and regulatory precedent through the FDA ISTAND program [40]. Liver-PBPK integration should serve as a template for expansion to other organ systems.

Third, strong validation practices characterize the field, with 95.8% of studies discussing clinical data comparisons and widespread use of quantitative metrics (AFE 77.5%, R^2^ 40.8%). These practices align with regulatory expectations for PBPK model qualification.

Fourth, substantial research gaps exist across organ-model combinations, with only liver-PBPK and liver-PK qualifying as established areas. Priority expansion targets include kidney, lung, and brain MPS-computational integration, as well as multi-organ systems with corresponding multi-compartment models.

Fifth, the evolution from single-organ to multi-organ MPS systems, which accelerated after 2021, reflects growing recognition that organ-organ interactions are essential for accurate pharmacokinetic prediction and the development of whole-body digital twins.

Sixth, the regulatory environment is increasingly favorable, with the FDA Modernization Act 2.0 (2022) [38], ISTAND program acceptance (2025) [40], and Roadmap to Reducing Animal Testing (2025) [39] signaling readiness to incorporate MPS-derived data into drug development decisions.

Seventh, although economic and translational benefits are widely acknowledged in qualitative terms, no studies have performed formal cost-effectiveness or cost-benefit analyses, representing a critical gap for demonstrating value to pharmaceutical decision-makers and justifying institutional adoption.

In summary, MPS-digital twin integration has transitioned from an emerging concept to a maturing research paradigm with demonstrated translational value. The field is well-positioned to address the pharmaceutical industry’s productivity crisis by providing human-relevant experimental data integrated with predictive computational models. Continued expansion across organ systems, standardization of methodologies, and demonstration of regulatory utility will be essential for realizing the full potential of this transformative approach to drug development.

## Footnotes

1
https://www.crd.york.ac.uk/PROSPERO/view/CRD420251274941
