# Supplementary material for: Integration of microphysiological systems with computational and digital twin modeling for pharmaceutical development: a systematic review
Source: J Pharm Pharm Sci. 2026 Sep 4;29:16454. doi: 10.3389/jpps.2026.16454 (PMC13585699; doi:10.3389/jpps.2026.16454)
Supplement: Supplementary file 1 [file Table1.docx]

**Supplementary Table S1. Software and Computational Tools Identified in Full-Text Analysis of MPS-Digital Twin Integration Studies.** Tools were identified through automated text-mining of 120 full-text documents. Software is organized by functional category. Individual tools are listed with their primary use category, the number of studies that mention the tool (n), and the percentage of the 120 analyzed studies. The Software Category Summary section shows aggregate counts by category; percentages exceed 100% because studies frequently employed multiple tools across categories. Detection identifies mentions of terminology within full-text documents, including methodological discussions and literature references, in addition to the primary use.

| **Software/Tool** | **Category** | **n** | **% of Studies** |
| --- | --- | --- | --- |
| ***PBPK/PK Modeling Software*** |  |  |  |
| MATLAB | General modeling | 24 | 20.0 |
| Simcyp | PBPK | 8 | 6.7 |
| GastroPlus | PBPK | 8 | 6.7 |
| PK-Sim/Open Systems Pharmacology | PBPK | 7 | 5.8 |
| Phoenix WinNonlin | PK/NCA | 7 | 5.8 |
| SimBiology | Systems pharmacology | 5 | 4.2 |
| Berkeley Madonna | ODE solver | 1 | 0.8 |
| ***Population PK Software*** |  |  |  |
| NONMEM | PopPK | 2 | 1.7 |
| Monolix | PopPK | 2 | 1.7 |
| Nlme | PopPK | 2 | 1.7 |
| ***Programming Languages*** |  |  |  |
| Python | General purpose | 12 | 10.0 |
| R | Statistical computing | 13 | 10.8 |
| Julia | Scientific computing | 2 | 1.7 |
| ***Statistical Software*** |  |  |  |
| GraphPad Prism | Statistics/graphing | 52 | 43.3 |
| SAS | Statistics | 18 | 15.0 |
| SPSS | Statistics | 8 | 6.7 |
| Stata | Statistics | 4 | 3.3 |
| ***Software Category Summary*** |  |  |  |
| Statistics Software (total) | — | 104 | 86.7 |
| MATLAB/SimBiology (total) | — | 30 | 25.0 |
| PBPK Software (total) | — | 25 | 20.8 |
| Python/Julia (total) | — | 14 | 11.7 |
| R (total) | — | 13 | 10.8 |
| PopPK Software (total) | — | 12 | 10.0 |

*Abbreviations: PBPK, physiologically-based pharmacokinetic; PK, pharmacokinetic; NCA, noncompartmental analysis; PopPK, population pharmacokinetics; ODE, ordinary differential equation.*
