# Supplementary material for: Integration of microphysiological systems with computational and digital twin modeling for pharmaceutical development: a systematic review
Source: J Pharm Pharm Sci. 2026 Sep 4;29:16454. doi: 10.3389/jpps.2026.16454 (PMC13585699; doi:10.3389/jpps.2026.16454)
Supplement: Supplementary file 2 [file Table2.docx]

**Supplementary Table S2. Commercial and Custom MPS Platforms Identified in Full-Text Analysis of MPS-Digital Twin Integration Studies.** Platforms were identified through automated text-mining of 120 full-text documents. For each platform, the table lists the manufacturer, primary product lines, key technical features, number of mentions across studies (n), and percentage of the 120 analyzed studies. Mention counts represent text-mining detection of platform-related terminology, which may include citations, methodological comparisons, or discussion of platform capabilities in addition to direct experimental use. Percentages exceed 100% because individual studies frequently referenced or utilized multiple platforms. Custom/Academic platforms are in-house-designed devices fabricated primarily using PDMS soft lithography.

| **Platform** | **Product Line(s)** | **Key Features** | **Mentions (n)** | **% of Studies** |
| --- | --- | --- | --- | --- |
| **Emulate** | Organ-Chip (Liver-Chip, Lung-Chip, Intestine-Chip, Kidney-Chip, Brain-Chip) | PDMS-based; mechanical stretch; vascular-epithelial interface; Liver-Chip S1 accepted into FDA ISTAND pilot program (qualification in progress) | 67 | 55.8 |
| **Mimetas** | OrganoPlate (2-lane, 3-lane, Graft) | Membrane-free; 96-well plate format; gravity-driven perfusion; high-throughput | 28 | 23.3 |
| **CN Bio Innovations** | PhysioMimix (Single-organ, Multi-organ) | Open-well; pneumatic pumping; primary hepatocyte culture; multi-organ linking | 23 | 19.2 |
| **TissUse** | HUMIMIC (Chip2, Chip3, Chip4) | Multi-organ; on-chip micro-pumps; ADME profiling; 28-day culture | 21 | 17.5 |
| **InSphero** | Akura Flow, Akura Viva, 3D InSight | Scaffold-free 3D microtissues; hanging drop technology; liver and pancreas models | 20 | 16.7 |
| **Hesperos** | Human-on-a-Chip | Multi-organ; functional readouts (MEA); neuromuscular junction; cardiac models | 9 | 7.5 |
| **Nortis** | Nortis Chip | Kidney tubule-on-chip; renal proximal tubule models; drug transport studies | 8 | 6.7 |
| **Custom/Academic** | In-house designed platforms | PDMS soft lithography; application-specific design; novel configurations | 89 | 74.2 |

*Abbreviations: PDMS, polydimethylsiloxane; ADME, absorption, distribution, metabolism, and excretion; MEA, microelectrode array; ISTAND, Innovative Science and Technology Approaches for New Drugs.*
