# Supplementary material for: Integration of microphysiological systems with computational and digital twin modeling for pharmaceutical development: a systematic review
Source: J Pharm Pharm Sci. 2026 Sep 4;29:16454. doi: 10.3389/jpps.2026.16454 (PMC13585699; doi:10.3389/jpps.2026.16454)
Supplement: Supplementary file 3 [file Table3.docx]

**Supplementary Table S3. Manual classification of machine learning / artificial intelligence studies.** This table presents the results of a manual re-screen of all studies in which machine learning or artificial intelligence was identified in the corpus. Each study was examined to determine the role of machine learning, whether the model was trained on MPS-derived data or external data, and an overall classification. The classification distinguishes predictive models trained on MPS data from image-analysis tools, review or perspective articles, in silico analyses without a trained model, and studies unrelated to MPS-model integration. Abbreviations: CIPN, chemotherapy-induced peripheral neuropathy; DDI, drug-drug interaction; ML, machine learning; MPS, microphysiological system; vMPS, vascularized microphysiological system.

| **Study (first author, year)** | **PMID** | **Role of ML/AI** | **Training data** | **Classification** |
| --- | --- | --- | --- | --- |
| Tronolone, 2023 | 38023704 | Neural network predicting oxygen transport | MPS-derived (vMPS morphology) | Predictive ML on MPS data |
| Ahmad, 2023 | 37033977 | Computer-vision tracking of leukocyte trafficking | MPS imaging | Image-analysis tool |
| Sun, 2025 | 40057746 | Deep-learning Cell Painting scoring for screening | MPS/cell imaging | Image-analysis tool |
| Matsuda, 2023 | 37888698 | Morphological deep learning for neurotoxicity (CIPN) | MPS imaging | Image-analysis tool |
| Ziemba, 2025 | 41303685 | Discussion of in silico/ML cytotoxicity testing | N/A | Review/perspective |
| Rodrigues, 2025 | 40671325 | Review of PBPK drug-interaction modeling | N/A | Review/perspective |
| Biondi-Zoccai, 2025 | 40763280 | Review of human-based cardiovascular technologies | N/A | Review/perspective |
| Kanukolanu, 2025 | 40945385 | Review of DDI prediction strategies | N/A | Review/perspective |
| Kulkarni, 2021 | 34000943 | Review of drug-induced kidney injury prediction | N/A | Review/perspective |
| Sillé, 2025 | 41000410 | Perspective proposing AI-calibrated digital twins | N/A | Review/perspective |
| Liu, 2024 | 38259971 | In silico/omics biomarker identification | Mixed | In silico support, no trained model |
| Solanki, 2023 | 37856428 | Neural-network computation on DNA/microfluidics | N/A | Off-topic (no MPS-model integration) |

*Of the twelve studies in which machine learning or artificial intelligence was identified, one applied a predictive model trained on MPS-derived data, three used machine learning as an image-analysis tool, six were review or perspective articles, one used in silico and omics analysis without a trained model, and one concerned an unrelated computational application.*
